# Epigenetics meets proteomics in an epigenome-wide association study with circulating blood plasma protein traits

**DOI:** 10.1038/s41467-019-13831-w

**Published:** 2020-01-03

**Authors:** Shaza B. Zaghlool, Brigitte Kühnel, Mohamed A. Elhadad, Sara Kader, Anna Halama, Gaurav Thareja, Rudolf Engelke, Hina Sarwath, Eman K. Al-Dous, Yasmin A. Mohamoud, Thomas Meitinger, Rory Wilson, Konstantin Strauch, Annette Peters, Dennis O. Mook-Kanamori, Johannes Graumann, Joel A. Malek, Christian Gieger, Melanie Waldenberger, Karsten Suhre

**Affiliations:** 1 Department of Physiology and Biophysics, Weill Cornell Medicine-Qatar, Doha, Qatar; 2 Computer Engineering Department, Virginia Tech, Blacksburg, VA USA; 3 Research Unit of Molecular Epidemiology, Helmholtz Zentrum München, German Research Center for Environmental Health, Neuherberg, Bavaria Germany; 4 Institute of Epidemiology, Helmholtz Zentrum München, German Research Center for Environmental Health, Neuherberg, Bavaria Germany; 5 German Centre for Cardiovascular Research (DZHK), partner site Munich Heart Alliance, Munich, Germany; 6 Proteomics Core, Weill Cornell Medicine‐Qatar, Doha, Qatar; 7 Genomics Core, Weill Cornell Medicine-Qatar, Doha, Qatar; 8 Institute of Human Genetics, Helmholtz Zentrum München, German Research Center for Environmental Health, Neuherberg, Germany; 9 Institute of Human Genetics, Technical University Munich, Munich, Germany; 10 Institute of Genetic Epidemiology, Helmholtz Zentrum München, German Research Center for Environmental Health, Neuherberg, Germany; 11 Chair of Genetic Epidemiology, IBE, Faculty of Medicine, LMU Munich, Munich, Germany; 12 Department of Clinical Epidemiology, Leiden University Medical Centre, Leiden, The Netherlands; 13 Scientific Service Group Biomolecular Mass Spectrometry, Max Planck Institute for Heart and Lung Research, W.G. Kerckhoff Institute, Bad Nauheim, Germany; 14 German Centre for Cardiovascular Research (DZHK), partner site Rhine-Main, Max Planck Institute of Heart and Lung Research, Bad Nauheim, Germany

**Keywords:** Proteomics, DNA methylation, Epigenomics, Quantitative trait

## Abstract

DNA methylation and blood circulating proteins have been associated with many complex disorders, but the underlying disease-causing mechanisms often remain unclear. Here, we report an epigenome-wide association study of 1123 proteins from 944 participants of the KORA population study and replication in a multi-ethnic cohort of 344 individuals. We identify 98 CpG-protein associations (pQTMs) at a stringent Bonferroni level of significance. Overlapping associations with transcriptomics, metabolomics, and clinical endpoints suggest implication of processes related to chronic low-grade inflammation, including a network involving methylation of *NLRC5*, a regulator of the inflammasome, and associated pQTMs implicating key proteins of the immune system, such as CD48, CD163, CXCL10, CXCL11, LAG3, FCGR3B, and B2M. Our study links DNA methylation to disease endpoints via intermediate proteomics phenotypes and identifies correlative networks that may eventually be targeted in a personalized approach of chronic low-grade inflammation.

## Abstract (web summary)

DNA methylation is associated with complex traits and the expression of genes and proteins. Here, Zaghlool et al. perform epigenome-wide association studies for 1,123 plasma proteins, replicate obtained protein (p)QTMs in an independent cohort and find overlap of pQTMs with expression QTMs and previously reported disease associations.

## Introduction

Genome-wide association studies (GWAS) with clinically relevant intermediate traits, such as gene expression^1^, proteomics^2^, and metabolomics^3^, unraveled numerous pathophysiological pathways and generated many hypotheses regarding the functional basis of complex disorders^4^. More recently, similar approaches linked variation in epigenetic modifications, especially differential methylation of chromosomal CpG-pairs, to changes in gene expression^5^ and blood circulating metabolites^6^.

DNA CpG methylation has been associated with many complex disorders, but the underlying pathophysiological processes often remain unclear. Changes in DNA methylation may be both, causal for changes in biological processes by differentially regulating gene expression, and consequential in response to modified physiological needs and disease. Blood circulating proteins as intermediate phenotypes can reveal the underlying disease-causing pathways. However, except for a study on a limited set of proteins^7^, no epigenome-wide association study (EWAS) with proteomics has been reported so far.

Unlike GWAS, where genetic variants can be assumed to be causal for the observed associations, the situation is more complex in EWAS. While changes in CpG methylation have been shown to induce changes in gene expression that can lead to changes in protein and metabolite levels, differences in transcript, protein, and metabolite levels, can in turn also induce changes in CpG methylation. Changes in the methylation of gene regulatory sites are generally driven by environmental challenges or by the body’s needs to cope with physiological dysregulations and disease.

A large fraction of the proteins studied here, many of them related to the immune system, are primarily produced by white blood cells. In these cases, a direct causal link between DNA methylation and circulating protein levels through regulation of white blood cell gene expression is possible. In cases where a physiological challenge affects the entire body and requires similar responses from all cells, DNA methylation measured in blood cells can constitute a proxy for processes occurring predominantly in other body tissues. For instance, Wahl et al.^8^ showed that methylation of obesity-associated CpG sites in blood cells and adipose tissues correlate. Age^9^, sex^10^, lifestyle^11^, disease state^12^, environmental factors^13^, cell type composition^14^, and genetic variations^5^ are all factors that have been shown to determine gene regulation and to induce differential CpG methylation in the process. It is therefore essential to account for these driving and potentially confounding factors in an EWAS approach. Complex networks of (co-)associated multi-omics traits, connecting CpG methylation, gene expression, protein, and metabolite levels to disease endpoints then emerge from such EWASs, as we recently showed at the example of a multi-omics association study with a small set of CpG sites^15^. Such networks might eventually guide a more personalized treatment of complex disorders, using for instance DNA methylation as a precise read-out of the body’s disease status with respect to the affected pathways, or to identify drug targets that may allow the modification of the underlying dysregulated processes.

Here, we report an EWAS of the human blood plasma proteome (pEWAS) with full replication. Motivated by previous EWAS with metabolomics^6^, the overarching goal of this study is to integrate methylation and proteomics data and to investigate the role of DNA methylation in disease. We quantify 1123 blood circulating proteins using the SOMAscan affinity proteomics platform (Somalogic Inc.)^2^ and determine the methylation levels of 470,837 CpG di-nucleotide sites using the Illumina Infinium 450K array (Illumina Inc.) in samples from 944 individuals of the population-based KORA (Cooperative Health Research in the Region of Augsburg) study^16^ and from 344 participants of the multi-ethnic Qatar Metabolomics Study on Diabetes (QMDiab)^17^ for replication.

**Fig. 1:**
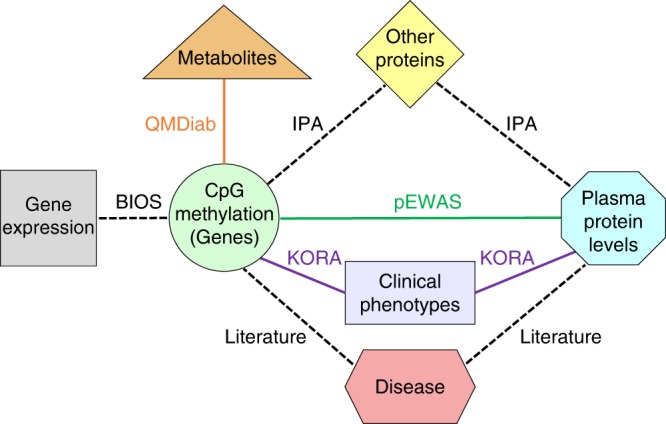
Study design and data integration. We conducted an EWAS with proteomics in KORA and replicated the associations in QMDiab (see Fig. 2). We used Ingenuity Pathway Analysis (IPA) to connect CpG-linked genes to their associated proteins through literature-reported observations and complemented the network with associations to gene expression (BIOS), metabolomics (QMDiab), and clinical endpoints (KORA), finally adding all previously reported disease associations. The resulting networks, using the same color code as here, are presented in Figs. 3 and 4.

To identify and isolate the general association-driving factors, we iteratively regress out CpG-protein associations that are driven by sex, blood cell composition, genetic variation, age, smoking, BMI, and diabetes. Using principal component analysis, we then confirm that no further global drivers remain. Next, we overlay our associations with CpG-gene expression associations (eQTMs) from the BIOS study^5^ and connect the CpG sites and the associated proteins using established experimental literature findings (Ingenuity Pathway Analysis, Qiagen)^18^. Finally, we complement our analysis with overlapping CpG- and protein-associations with blood, urine, and salivary metabolites (QMDiab), and with complex disease endpoints (KORA), revealing individual sites and multi-omics networks that connect associations between CpG methylation and disease-relevant endpoints through blood circulating proteins (Fig. 1).

## Results

### A step-wise EWAS with proteomics identified 12,606 pQTMs

**Fig. 2:**
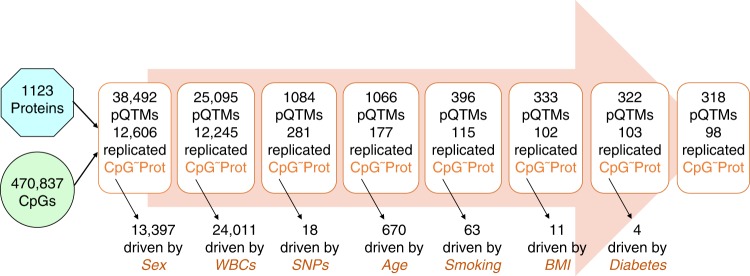
Summary of the step-wise EWAS. A series of pEWASs was carried out in the discovery cohort KORA, followed by replication in the QMDiab study (Supplementary Data 2 and 3). In the initial step, methylation levels of 470,837 CpGs (M-values, winsorized) were tested for association with 1123 blood circulating protein concentrations (log-scaled, winsorized), leading to 38,492 associations that reached stringent Bonferroni significance, 12,606 of which were replicated. Potential driving factors (sex, white blood cell composition, genetic variants, age, smoking, BMI, and diabetes) were successively regressed out from the CpG and the protein levels, using the residues in the next EWAS step (see Methods). At each step, a number of associations (pQTMs) fell below the significance threshold (indicated by the black arrows). These associations were likely driven by the factor used in the previous regression step. Eventually, 318 pQTMs remained that were not driven by any of the factors listed here, 98 of which were replicated (Table 1).

After stringent quality control, we analyzed 470,837 CpG sites for epigenome-wide associations with 1123 protein levels (Supplementary Data 1), determined in 944 blood samples from the KORA study (see Methods). We identified 38,492 associations between CpG methylation and blood circulating protein levels (pQTMs), using a conservative Bonferroni level of significance (*P* < 0.05/470,837/1123 = 9.46 × 10^−11^). We then iteratively and separately regressed the methylation and proteomics data on potential drivers, starting with sex, followed by blood cell composition, genetic variation, age, smoking, BMI, and diabetes (Fig. 2 and Supplementary Data 2). Finally, we computed the first 10 principal components (PCs) of the methylation and protein levels and asked whether any of these PCs associated with any of the protein or CpG methylation levels, respectively. None of these PCs accounted for more than 1.5% of the explained variance in the methylation or the protein levels, and further regressing them out did not change the remaining pQTM list. Hence, we did not include any PCs into the final regression.

At each step, we attempted replication of the identified pQTMs in the QMDiab study, where we applied an identical step-wise regression approach (Supplementary Data 3). We considered a pQTM as replicated if it reached a Bonferroni level of significance (*P* < 0.05/N, N being the number of pQTMs identified at the respective discovery step). In all, 12,606 of the 38,492 uncorrected pQTMs replicated in QMDiab, implicating 10,047 CpG sites and 116 proteins. The most important drivers of these associations were white blood cell composition and sex, followed by age and smoking. After regressing out all identifiable driving factors, 318 pQTMs remained, out of which 98 replicated in QMDiab (Tables 1–3, Fig. 2, Supplementary Fig. 1; Supplementary Data 4). The Manhattan plots of the replicated pQTMs and histograms of the genomic inflation rates for each step are provided in Supplementary Figs. 2 and 3.

**Table 1:** Replicated PAPPA pQTMs.

CpG	pQTM (this study)	*P*_pQTM_	Beta_pQTM_	eQTM (BIOS)	*P*_eQTM_	Beta_eQTM_
cg07708453 (*PRDM2*) chr1:14,032,034	PAPPA (4148-49_2) chr9:118,916,083-119,164,601	3.10 × 10^−16^	0.262	ENSG00000116731 PRDM2	2.52 × 10^−6^	0.130
cg19393755 (*CPSF4L*) chr17:71,258,101	PAPPA	2.03 × 10^−14^	−0.246	ENSG00000179604 CDC42EP4^a^	3.58 × 10^−11^	0.132
cg10831642 (*SH3PXD2A*) chr10:105,378,344	PAPPA	8.19 × 10^−12^	−0.246	ENSG00000107957 SH3PXD2A	9.03 × 10^−32^	0.287
cg26272069 (*GABBR1*) chr6:29,591,706	PAPPA	9.25 × 10^−12^	−0.224	ENSG00000204681 GABBR1	3.80 × 10^−8^	−0.073
cg20290167 (*METRNL*) chr17:81,040,724	PAPPA	5.58 × 10^−11^	−0.212	ENSG00000176845 METRNL	2.77 × 10^−5^	0.091
–	(Total: 72 PAPPA pQTMs)	–	–	n.a.	n.a.	n.a.

The *p*-value (*P*_pQTM_, linear regression) and regression coefficient (Beta_pQTM_) from the discovery study are reported. The chromosomal position of the CpG sites and the related protein coding genes are given, together with the associated protein and SOMAmer identifiers (SeqId). The BIOS QTL server^5^ was used to identify overlapping CpG methylation to gene expression associations (eQTMs). The respective *p*-values (*p*_eQTM_, linear regression) and regression coefficients (beta_eQTM_) of the association of the respective CpG and the transcript are reported. All associations are located in trans, that is, the CpG and the blood circulating protein coding region were >1 Mbp. The five selected pQTMs here are only those associated with an overlapping eQTM. The full list is provided in Supplementary Data 4

^a^These genes belong to the same cytogenic band as those reported by Illumina as being regulated by the respective CpG sites and are within physical proximity (<21,000 and 5000  bp downstream, respectively)

**Table 2:** Replicated NLRC5 pQTMs.

CpG	pQTM (this study)	*P*_pQTM_	Beta_pQTM_	eQTM (BIOS)	*P*_eQTM_	Beta_eQTM_
cg07839457 (*NLRC5*) chr16:57,023,022	CD48 (3292-75_1) chr1:160,648,536-160,681,641	1.10 × 10^−21^	−0.306	n.a.	n.a.	n.a.
cg08159663 (*NLRC5*) chr16: 57,022,486	CD48	3.38 × 10^−12^	−0.246	ENSG00000140853 NLRC5	8.26 × 10^−10^	0.213
cg07839457 (*NLRC5*) chr16:57,023,022	B2M (3485-28_2) chr15:45,003,675-45,011,075	7.60 × 10^−20^	−0.292	n.a.	n.a.	n.a.
cg16411857 (*NLRC5*) chr16:57,023,191	B2M	1.94 × 10^−15^	−0.256	n.a.	n.a.	n.a.
cg00218406 (*HCP5*) chr6:31,431,407	B2M	3.97 × 10^−13^	−0.234	ENSG00000206337 HCP5	7.78 × 10^−102^	0.454
cg08099136 (*PSMB8*) chr6:32,811,251	B2M	4.80 × 10^−11^	−0.214	ENSG00000204264 PSMB8	1.96 × 10^−23^	0.258
cg07839457 (*NLRC5*) chr16:57,023,022	CXCL10 (4141-79_1) chr4:76,942,273-76,944,650	8.06 × 10^−19^	−0.283	n.a.	n.a.	n.a.
cg07839457 (*NLRC5*) chr16:57,023,022	FCGR3B (3311-27_1) chr1:161,592,986-161,601,753	1.02 × 10^−18^	−0.307	n.a.	n.a.	n.a.
cg08159663 (*NLRC5*) chr16:57,022,486	FCGR3B	1.44 × 10^−14^	−0.290	ENSG00000140853 NLRC5	8.26 × 10^−10^	0.213
cg16411857 (*NLRC5*) chr16:57,023,191	FCGR3B	3.79 × 10^−13^	−0.260	n.a.	n.a.	n.a.
cg07839457 (*NLRC5*) chr16:57,023,022	LAG3 (5099-14_3) chr12:6,881,678-6,887,621	1.29 × 10^−17^	−0.274	n.a.	n.a.	n.a.
cg08159663 (*NLRC5*) chr16:57,022,486	LAG3	9.22 × 10^−13^	−0.246	ENSG00000140853 NLRC5	8.26 × 10^−10^	0.213
cg07839457 (*NLRC5*) chr16:57,023,022	CD163 (5028-59_1) chr12:7,623,409-7,656,489	1.83 × 10^−15^	−0.256	n.a.	n.a.	n.a.
cg07839457 (*NLRC5*) chr16:57,023,022	CXCL11 (3038-9_2) chr4:76,954,835-76,962,568	1.13 × 10^−13^	−0.246	n.a.	n.a.	n.a.

The *p*-value (*P*_pQTM_, linear regression) and regression coefficient (Beta_pQTM_) from the discovery study are reported. The chromosomal position of the CpG sites and the related protein coding genes are given, together with the associated protein and SOMAmer identifiers (SeqId). The BIOS QTL server^5^ was used to identify overlapping CpG methylation to gene expression associations (eQTMs). The respective p-values (p_eQTM_, linear regression) and regression coefficients (beta_eQTM_) of the association of the respective CpG and the transcript are reported. All associations are located in trans, that is, the CpG and the blood circulating protein coding region were < 1 Mbp

**Table 3:** Other replicated pQTMs.

CpG	pQTM (this study)	*P*_pQTM_	Beta_pQTM_	eQTM (BIOS)	*P*_eQTM_	Beta_eQTM_
cg10604476 (*ICAM5*) chr19:10,403,908	ICAM5 (5124-69_3) chr19:10,400,657-10,407,454	6.09 × 10^−25^	0.356	ENSG00000105376 ICAM5	2.22 × 10^−9^	0.186
cg03650189 (*ICAM5*) chr19:10,405,083	ICAM5	1.22 × 10^−24^	0.344	ENSG00000105376 ICAM5	1.68 × 10^−5^	0.129
cg22910295 (*ICAM5*) chr19:10,403,862	ICAM5	3.72 × 10^−24^	0.339	ENSG00000105376 ICAM5	2.37 × 10^−6^	−0.134
cg15011409 (*ICAM5*) chr19:10,405,226	ICAM5	5.96 × 10^−23^	0.331	ENSG00000105376 ICAM5	2.66 × 10^−12^	0.198
cg21994045 (*ICAM5*) chr19:10,403,936	ICAM5	4.18 × 10^−17^	0.291	ENSG00000105376 ICAM5	1.21 × 10^−5^	−0.148
cg10773601 (*CLEC11A*) chr19:51,226,046	CLEC11A (4500-50_2) chr19:51,226,586-51,228,974	8.06 × 10^−27^	−0.341	ENSG00000105472 CLEC11A	1.06 × 10^−71^	0.424
cg16651537 (*CLEC11A*) chr19:51,226,536	CLEC11A	1.67 × 10^−24^	−0.326	ENSG00000105472 CLEC11A	3.54 × 10^−63^	0.434
cg05575921 (*AHRR*) chr5:373,378	PIGR (3216-2_2) chr1:207,101,863-207,119,811	8.08 × 10^−16^	−0.264	ENSG00000180104 EXOC3^a^	1.19 × 10^−6^	0.063
cg18419358 (n.a.) chr6:158,384,009	GP1BA (4990-87_1) chr17:4,835,592-4,838,325	2.52 × 10^−12^	0.228	n.a.	n.a.	n.a.
cg27535410 (*PRTN3*) chr19:846,354	PRTN3 (3514-49_2) chr19:840,963-848,175	1.21 × 10^−11^	−0.219	n.a.	n.a.	n.a.
cg13028630 (*C4B*/*C4A*) chr6:31,964,754	C4A/C4B (4481-34_2) chr6:31,937,353-32,079,643	1.38 × 10^−11^	−0.246	n.a.	n.a.	n.a.
cg09488502 (*SIGLEC5*) chr19:52,134,289	SIGLEC14 (5125-6_3) chr19:52,145,806-52,150,054	4.89 × 10^−11^	0.220	n.a.	n.a.	n.a.

The *p*-value (*P*_pQTM_, linear regression) and regression coefficient (Beta_pQTM_) from the discovery study are reported. The chromosomal position of the CpG sites and the related protein coding genes are given, together with the associated protein and SOMAmer identifiers (SeqId). The BIOS QTL server^5^ was used to identify overlapping CpG methylation to gene expression associations (eQTMs). The respective *p*-values (*p*_eQTM_, linear regression) and regression coefficients (beta_eQTM_) of the association of the respective CpG and the transcript are reported. Some of the associations are located in cis, that is, the CpG and the blood circulating protein coding region were within 1 Mbp (e.g. CLEC11A and ICAM5), while others were located in trans (e.g. AHRR)

^a^These genes belong to the same cytogenic band as those reported by Illumina as being regulated by the respective CpG sites and are within physical proximity (<21,000 and 5000 bp downstream, respectively)

### Genomic enrichment

After carrying out genomic enrichment at all EWAS steps, we found that enrichment/depletion is for the most part consistent across all regression steps (Supplementary Data 5). Among the 10,047 CpG sites included in the 12,606 replicated pQTMs, we noticed a substantial 2.12-fold enrichment in genomic enhancers regions (*p* = 2.72 × 10^−259^) and a strong 0.30-fold depletion (*p* = 3.00 × 10^−276^) in promoter associated CpG sites. Cell type specific promoter associated CpG sites, in contrast, were enriched 1.90-fold (*p* = 1.01 × 10^−18^) (Supplementary Fig. 4). We also checked the 10,047 CpG sites involved in the pQTMs for overlap with intra- and inter-chromosomal contact using high-resolution Hi-C data, a method that probes the three-dimensional architecture of whole genomes by coupling proximity-based ligation with massively parallel sequencing^19^. For these CpG sites, we found a 1.73-fold enrichment for Hi–C inter-chromosomal contact (*p* = 2.44 × 10^−81^) and a 1.79-fold enrichment for Hi–C intra-chromosomal contact (*p* = 8.27 × 10^−112^). As for the 98 final replicated pQTMs, we also found enrichment in mapping to active regions. For instance, enhancers were enriched by a 2.90-fold (*p* = 6.54 × 10^−7^), and promoter associated CpG sites were depleted by a 0.59-fold (*p* = 9.00 × 10^−3^). All other tested CpG characterizations were not significant in the 98 final pQTMs due to limited statistical power, although the directions of enrichment/depletion were still coherent with what was observed in the previous steps.

### 98 pQTMs have no common biological driver

**Fig. 3:**
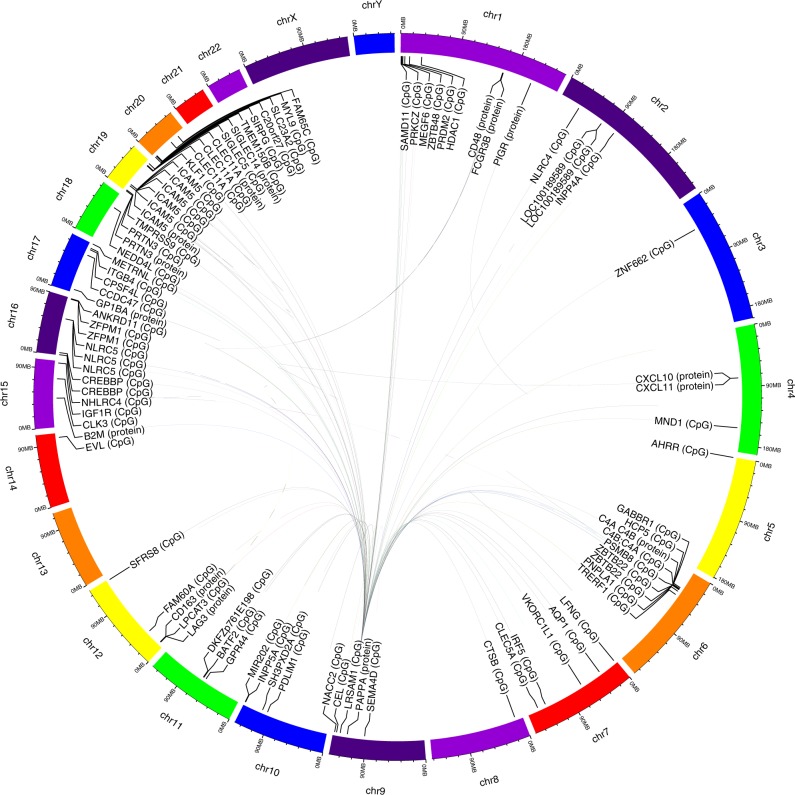
The methylation-proteome network. Circular plot of all 98 replicated cis- and trans- pEWAS associations.

The 98 replicated pQTMs comprised 89 unique CpGs and 15 unique proteins. Ten of the 98 pQTMs were cis-pQTMs, that is, the gene coding for the associated protein was encoded within 1 Mb of the CpG site. The 98 pQTMs could be grouped into nine independent association signals, which we labeled PAPPA, NLRC5, CLEC11A, ICAM5, C4, SIGLEC5, PRTN3, AHRR, and GP1BA, in reference to the respective characterizing CpG gene locus or associated protein (Tables 1–3 and Fig. 3). The largest group comprised 72 pQTMs which all involved associations with PAPPA at 70 independent genetic loci (trans-pQTMs) (Table 1). The second largest group of pQTMs, labeled NLRC5, involved three CpG sites (*NLRC5*, *HCP5*, and *PSMB8*) that associated with one or several of seven proteins of the immune system (CD48, CD163, CXCL10, CXCL11, LAG3, FCGR3B, and B2M) (Table 2). The remaining seven pQTM groups consisted of a single CpG locus, in two cases involving multiple correlated CpGs, which associated with a single protein. Two of these were trans-pQTMs (AHRR, GP1BA), the other five were cis-pQTMs (ICAM5, CLEC11A, PRTN3, C4, and SIGLEC5) (Table 3).

To identify the biological processes that may be related to these 98 pQTMs, we characterized the implicated CpG sites and proteins using additional data sets: Using the BIOS QTL server^5^ we identified 18 expression QTMs (eQTMs) that overlapped with the 89 unique CpG sites. Using the EWAS Atlas^20^, we identified 215 previously reported disease associations among numerous studies (Supplementary Data 6). We further tested the 89 unique CpG sites and the 15 unique proteins of the 98 pQTMs for association with various clinical phenotypes that were available in KORA, that is, hypertension, myocardial infarction, metabolic syndrome, type 2 diabetes, and other disease relevant clinical phenotypes, including body mass index, alcohol consumption, total cholesterol, HDL, LDL, and triglycerides. At a stringent Bonferroni level of significance (correction for number of proteins and clinical phenotypes), we identified associations for 14 of the proteins and eight of the CpG sites (Supplementary Data 7 and 8). Finally, we tested the 89 CpG sites for association with 2,251 urinary, salivary, and blood metabolites that we previously reported^15^. We found 20 associations at Bonferroni significance (*p* < 2.5 × 10^−7^ = 0.05/89/2251) (Supplementary Data 9). Thirteen of the metabolic associations were with the *AHRR* smoking locus (cg05575921). Furthermore, the strongest metabolic association for all 5 CpG sites pertaining to the *NLRC5* locus was with urinary 1,3,7-trimethylurate (*p* = 1.70 × 10^−19^–1.97 × 10^−9^, Supplementary Fig. 5), a metabolite that is a readout of caffeine metabolism and that is known to negatively regulate genes of the inflammasome, such as *NLRC4*^21^.

## Discussion

We have carried out a large epigenome-wide association study with proteomics data. A previous association study with protein levels investigated 121 protein biomarkers^7^ from the Olink platform, of which we replicated the essential associations on the overlapping marker set (Supplementary Note 1; Supplementary Data 10). Unlike GWAS, in which genetic variants remain constant over the course of an individual’s lifetime and can be assumed to be causal for the observed associations, EWAS are known to be confounded by many factors. As we show in this study, the vast majority of the associations between DNA methylation and protein levels are driven by factors that have been associated with DNA methylation before, such as sex^22^, blood cell composition^14^, genetic variation^5^, age^9^, smoking^11^, body mass index^23,24^ and diabetes^25^. Accordingly, many proteins that occur in pQTMs here, have also been previously found in association with these driving factors, including leading sex-associated proteins^26^, proteins associated with smoking^15^, and proteins that have been reported in a GWAS^2^, confirming the validity of the approach (see Supplementary Note 2 for details).

Interestingly, we observe a 0.30-fold depletion in promoter associated CpG sites, contrasted by a 1.90-fold enrichment in cell type specific promoter associated CpG sites, and an enrichment in CpG sites in enhancers and inter- and intra-chromosomal contact regions. These observations support the idea that these pQTMs represent readouts of important cellular regulatory processes, rather than reflecting random DNA methylation events. In particular, in cases where these CpG sites overlap with disease associations, our pEWAS associations add an additional level of understanding to the underlying pathophysiology, as they implicate the respective proteins by solid and replicated experimental evidence.

After elimination of all known general driving factors, 98 associations emerge that are likely to be driven by more specific biological processes. Of particular interest are the two larger groups, PAPPA and NLRC5, which we discuss in the following. 72 pQTMs are associations of PAPPA with CpG sites distributed across the genome. Methylation of the majority of these CpG sites (78%) correlated negatively with PAPPA levels (Supplementary Fig. 6). When combined into a single linear regression model, all 72 CpG sites account for a considerable 42.2% of the observed variance in PAPPA. On the individual protein level, the associations with PAPPA remain relatively inflated (lambda = 1.37), indicating that further CpG sites also associate with PAPPA to a certain extent, but not strongly enough to reach Bonferroni significance (Supplementary Fig. 3h). This suggests that PAPPA had still to be confounded by some global factor, possibly a cell type that the Houseman method did not account for. We therefore tested the experimental blood cell composition (measured only in QMDiab) for association with PAPPA and found a strong signal with eosinophil count. In fact, 70 out of the 72 pQTMs involving PAPPA were no longer significant in QMDiab after regressing out the eosinophil count. This suggests that the process driving the PAPPA associations likely occurs predominantly in eosinophils, a blood cell type that is often found elevated in allergic reactions^27^.

**Fig. 4:**
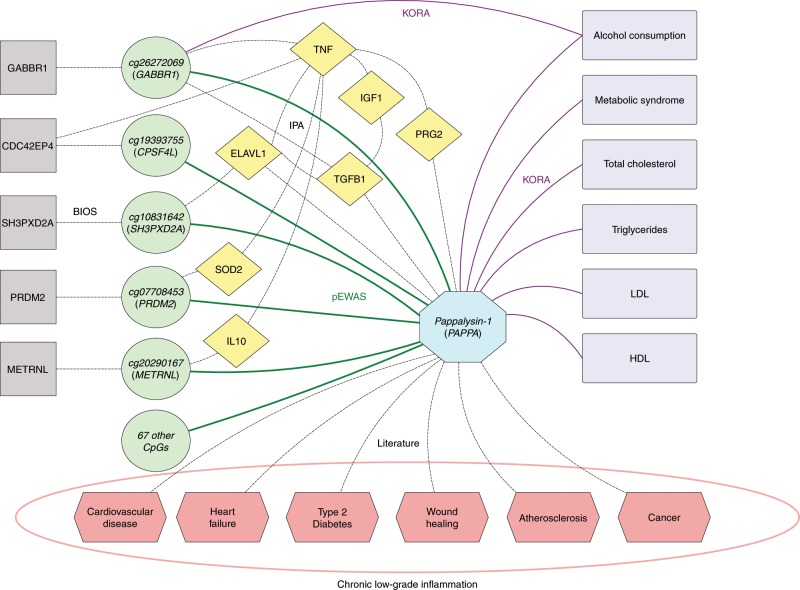
Pappalysin-1 network. This network comprises 72 CpG sites (green circles, same color and shape code as in Fig. 1) that associated with blood circulating levels of pappalysin-1 (PAPPA) (blue octagon), five of which were also associated with RNA expression in BIOS (gray squares) (Table 1). Ingenuity Pathway Analysis (IPA) was used to connect these CpG sites to PAPPA through protein-protein links (yellow diamonds) that were supported by experimental findings, reflecting the well documented role of PAPPA as an activator of the IGF and NFκB pathways. PAPPA levels also associated with relevant clinical phenotypes in KORA, reaching multiple testing corrected significance levels of *p* < 5.6 × 10^−4^ for CpG sites and *p* < 3.6 × 10^−3^ for proteins (purple rectangles). PAPPA has also been linked to various diseases in numerous previous studies (pink hexagons). Full literature support of these links is provided in Supplementary Note 3.

We used Ingenuity Pathway Analysis (IPA, Qiagen Inc.)^18^ to construct a network connecting selected genes, (ie. those having a transcript association in BIOS), to PAPPA (Fig. 4 and Supplementary Note 3). Pappalysin-1, or pregnancy-associated plasma protein-A, is a metalloproteinase that cleaves insulin-like growth factor binding proteins (IGFBPs) resulting in the activation of the insulin growth factor (IGF) pathway^28^. Although PAPPA has been discovered for its vital role in pregnancy, this protein acts as an oncogene, promoting tumor cell proliferation, invasion, and metastasis^29^. It is considered a marker of response to injury or diseases such as atherosclerosis or lesion progression^30^. One of the main proteolytic roles of PAPPA is the activation of the Nuclear Factor Kappa B (NFκB), phosphatidylinositol-3 (PI3K), Akt kinase (AKT), and extracellular-signal-regulated kinase (ERK) signaling pathways^29^. PAPPA also plays a role in bone formation, inflammation, wound healing, and female fertility^31^. High levels of PAPPA have been shown to be associated with increased risk of heart failure^32^. PAPPA also is a strong predictor for adverse cardiovascular events in patients with type 2 diabetes^33^ and preeclampsia^34^. Although the regulation of PAPPA has not been exhaustively studied, accumulating evidence shows that pro-inflammatory cytokines, such as TNF-α and IL-1β, are the main regulators of PAPPA expression in dermal fibroblasts, arterial endothelial cells and smooth muscle cells. In addition, IL-6 and transforming growth factor-beta (TGF-β) have also been shown to promote PAPPA expression^35^. Because there are three potential NFκB binding sites upstream of PAPPA, cytokine-induced PAPPA expression may be mediated by NFκB activation^36^. This makes PAPPA, which is widely expressed in multiple tissues, or any of its regulators, desirable therapeutic targets that may indirectly inhibit IGF signaling in tissues where this signaling is driven by increased PAPPA activity.

The second largest group (NLRC5) comprises 14 pQTMs located at three genetic loci with five associated CpGs (cg07839457, cg16411857, and cg08159663 near the transcription start site of the *NLRC5* locus on chromosome 16; cg00218406 at the *HCP5* locus and cg08099136 at the *PSMB8* locus on chromosome 6, the latter two CpGs are 1.4MB apart). These five CpGs associated with one or more of the following seven proteins: Beta-2-microglobulin (B2M), CD48 antigen (CD48), Low affinity immunoglobulin gamma Fc region receptor III-B (FCGR3B), Lymphocyte activation gene 3 protein (LAG3), Scavenger receptor cysteine-rich type 1 protein M130 (CD163), and C-X-C motif chemokines 10 and 11 (CXCL10 and CXCL11). The five CpG sites were all positively correlated with one another, as were the seven proteins; methylation of the CpG sites was anti-correlated with the protein levels (Supplementary Fig. 7).

**Fig. 5:**
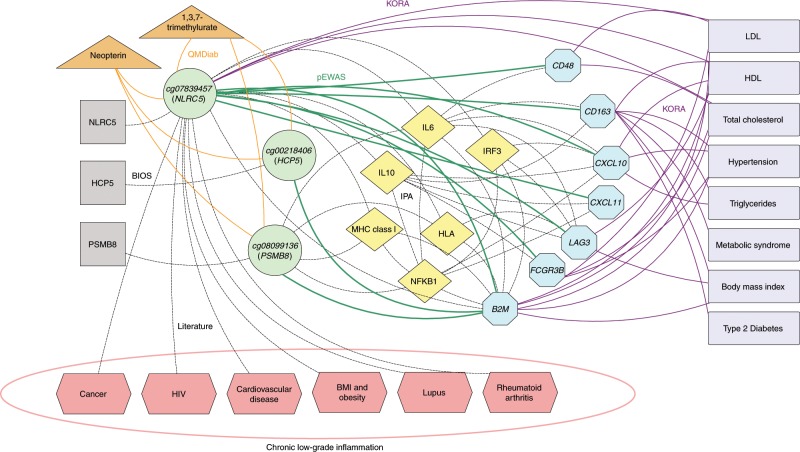
NLRC5 network. Our pEWAS (green lines) identified multiple proteins (blue octagons) and CpG sites (green circles) that are related to major anti-inflammatory pathways, and that could be directly connected via intermediate genes and proteins using IPA (yellow diamonds). *NLRC5* and beta-2-microglobulin both regulate the major histocompatibility complex MHC class I genes through interaction with various interleukins and NFκB. *NLRC5* methylation also associated with various metabolic inflammatory markers (orange triangles). Finally, the associated proteins were are also associated with various clinical phenotypes in KORA at multiple-testing corrected significance levels (purple rectangles). *NLRC5* methylation is a hallmark of chronic inflammation and has been reported in association with several inflammation-related diseases (pink hexagons). Full annotations of these links are provided in Supplementary Note 4.

Using IPA, we constructed a network connecting the genes that were putatively regulated by the associated CpG sites (*NLRC5*, *PSMB8*, and *HCP*) with the seven associated proteins (Fig. 5). The IPA search revealed the following relationships: *NLRC5* belongs to the NLR family and is a transcriptional regulator of MHC class genes through interaction with RFX transcription factor components. B2M is a component of the major histocompatibility complex (MHC) class I. MHC class I molecules play an important role in antigen presentation and immunoglobulin transport. *NLRC5* and B2M are directly linked: in humans, *NLRC5* increases the MHC class I expression of the B2M protein^37,38^. Indirect links were found between the CpG sites and the associated proteins through various Interleukins (IL-6 and IL-10), major histocompatibility complexes (HLA-A, HLA-abc, MHC Class I, and Interferon regulatory factor 3 (*IRF3*) (Supplementary Note 4). These intermediate molecules are all functionally linked to B2M and are also all related to NFκB), which is also linked to PSMB8, B2M, CXCL10, and CXCL11. NLRC5 is also a crucial negative regulator that blocks two major components of the NFκB and type I interferon pathways, serving as a central component in the homeostatic control of the innate immune system^39^. There is also a direct link between NFκB and B2M. NFκB and IRF3 are both known to strongly trans-activate B2M in human hematopoietic cells^40^. For a detailed description of all experimental findings supporting each connection in this network see Supplementary Note 4.

It is believed that NLRC5 limits the activation of inflammatory pathways^41^. Although NLRC5 is known to contribute to processes related to inflammatory type I interferon and adaptive immune responses beyond the regulation of MHC class I genes, these functions are currently not well understood and blurred by discrepancies in the reported data^42^. NLRC5 is known to be involved in the regulation of signal transduction pathways and pro-inflammatory cytokine production. Interleukin-18 and interleukin-1β are two of the key inflammatory cytokines. *NLRC5* methylation associated with circulating IL-18 levels and with cardiovascular disease^43^. In a previous EWAS with proteins measured using the Olink platform^7^, *NLRC5* methylation (cg07839457) was associated with CXCL9, CXCL11, IL-12, and IL-18 levels. The SOMAscan platform does not have a probe for IL-18, but interestingly, we observe an association between *NLRC5* methylation and interleukin-18-binding protein (IL18BP, *p* = 4.27 × 10^−5^). *NLRC5* methylation has also been reported in association with Soluble Tumor Necrosis Factor Receptor 2 (sTNFR2), a marker of cardiovascular disease risk in individuals with diabetes^44^. Two of the proteins in the NLRC5 network (CD163 and FCGR3B) were also linked to cardiovascular diseases. CD163 and FCGR3B were both linked to cardiovascular disorder of the myocardium and coronary heart disease. Upregulation of human CD163 protein in detergent-resistant membranes from human peripheral blood monocytes was associated with coronary artery disease and myocardial infarction^45^. NLRC5 is also known to be associated with Tumor Necrosis Factor-alpha (TNFα) which is a marker of systemic inflammation^46^. We observed several TNF associations with cg07839457 at a relaxed significance level (Supplementary Data 11). TNFα is one of the key players in the regulation of inflammatory responses and is a drug target in several cardiovascular diseases, including congestive heart failure and coronary artery disease^46^. A recent EWAS of circulating TNFα identified *NLRC5* methylation (cg16411857, cg07839457) as one of its three top hits and showed that *NLRC5* methylation was linked to gene expression and inversely associated with the risk of incident coronary heart disease^47^.

We also observed multiple associations of *NLRC5* methylation with urinary markers of inflammation. The strongest CpG-metabolite association was with 1,3,7-trimethylurate, a metabolite found to be altered in multiple sclerosis^48^, cerebral malaria^49^, and welding fume exposure^50^, all of which involve systemic inflammation processes. *NLRC5* methylation was further associated with urinary neopterin (*p* = 1.79 × 10^−7^) (Supplementary Fig. 5), an established metabolic marker of inflammation^51^ that has been observed elevated in urine from subjects with acute kidney allograft rejection. Neopterin is also a measure of macrophage activation^52^. *NLRC5* methylation at cg07839457 has been previously linked to numerous diseases. For instance, *NLRC5* methylation was associated with BMI and obesity in Africans^53^ and with HIV infection^54^. Hypo-methylation of *NLRC5* was also found to be strongly correlated with age in centenarians^55^. Furthermore, *NLRC5* methylation has been associated with lupus^44^ and rheumatoid arthritis^56^. Recently, NLRC5 has also taken a central role in tumor immunology^57^. Taken together, the robustly reported associations of *NLRC5* methylation with many chronic low-grade inflammation related diseases suggest that the connections we report here between the genes, proteins and metabolites of the NLRC5 network represent a coherent and comprehensive model of the pathways and processes that are jointly involved in all of these diseases.

The remaining pQTMs include ten cis-pQTMs (CLEC11A, ICAM5, PRTN3, C4, and SIGLEC5) which likely reflect the regulatory chain from DNA methylation through gene expression to protein levels. The driving factors behind these associations remain to be identified. The two trans-pQTMs (AHRR, GP1BA) also provide interesting insights. Despite regressing out smoking, *AHRR* (cg05575921) methylation remains associated with polymeric immunoglobulin receptor (PIGR). Notably, both PIGR and *AHRR* methylation were also associated with triglycerides in KORA (*p* = 2.01 × 10^−7^ and *p* = 6.17 × 10^−4^ respectively). Similarly, GP1BA associated with body mass index, metabolic syndrome, and hypertension in KORA (*p* = 6.66 × 10^−6^–3.72 × 10^−4^). This suggests that there may be some residual confounding, or additional unidentified environmental factors driving these associations.

We are aware of several limitations to our study. First, aptamer-based proteomics methods and array-based methylation assays are susceptible to potential probe cross-reactivity and non-specific binding^58^. However, we verified that none of the CpG sites or proteins that were involved in the final 98 pQTMs have been flagged for such issues (see Methods). Two of the fifteen proteins have been directly validated by mass spectrometry in plasma, and six were detected as enriched in various cells or serums in a previous study^59^. Second, DNA has been obtained from white blood cells and proteins were measured in plasma. Our study is hence bound to identify processes that occur primarily in blood, or that are general to the organism. EWAS conducted in other tissues and cell types are likely to reveal further associations with proteins that are not primarily controlled by white blood cells. Regarding our approach of successively regressing out confounders, although it is often very similar to an evaluation in which all influential variables are inserted in a single regression, it is not necessarily identical. Differences between the two approaches could for instance arise in the case of interactions between two predictors. We therefore verified that this was not the case for the pQTMs we report here (Supplementary Data 12).

In conclusion, we like to stress that it is essential to appreciate that the associations we reported here were obtained by analyzing samples from almost 1,300 individuals, reflecting experimental data obtained from naturally occurring variance of the general population where each individual may be viewed as an experiment conducted by Nature^8,60^. The underlying processes were hence equally present in Caucasians and robustly replicated in a multi-ethnic population, and therefore likely reflect important regulatory pathways that were present to a different extent in most individuals. The two large CpG-protein networks we reported here provide correlative evidence for interactions between the involved genes, proteins, and metabolites, and thereby contribute to a better understanding of the regulatory processes that are associated with chronic low-grade inflammation^61,62^.

## Methods

### Study population (KORA)

The KORA F4 study is a population-based cohort of 3,080 subjects living in the southern Germany. Study participants were recruited between 2006 and 2008 comprising individuals with age ranging from 32 to 81. All KORA participants have given written informed consent and the study was approved by the Ethics Committee of the Bavarian Medical Association. For this study, joint data was available for methylation, proteomics, and genotyping measurements of 944 individuals. The DNA methylation data set was determined using the Infinium HumanMethylation450 BeadChip platform which was described in detail previously^6^. Aptamer-based proteomics was done using the SOMAscan platform and has been described in detail elsewhere^2^. Genotyping was performed using the Affymetrix Axiom array, also described in detail elsewhere^2^.

### DNA methylation (KORA)

The KORA methylation dataset originally consisted of 1814 samples, of which 944 samples had both proteomics and genotyping measurements. The methylation dataset was preprocessed according to the following steps. Measurements from the 65 probes targeting SNPs (as identified in the Illumina manifest) were first excluded. Then background correction was performed using the R package minfi. Then the detection rate was examined and any samples having a detection rate below 95% were excluded. Normalization was then carried out on the intensity methylation values using the minfi R package, using a procedure equivalent to the Lumi:QN + BMIQ pipeline. Estimation of the white blood cell proportions was performed according to the Houseman method using 473 of the 500 most cell specific CpG sites that were present on the 450K array. Methylation measurements for 485,512 CpG sites were available. 35 CpG sites were omitted due to having fewer than 100 measurements and CpG sites present on the X and Y chromosomes (11,231 and 416 sites respectively) were also omitted from the analysis due to the remarkable bias they create in the pEWAS. Finally, 2993 non-cg CpG sites were also excluded from the dataset leaving 470,837 CpG sites for subsequent analysis.

### Proteomics (KORA)

The SOMAscan platform was used to quantify protein levels in undepleted plasma of 997 KORA individuals^26,61–68^. Briefly, samples were split into three dilution bins (0.05, 1, and 40%) and incubated with bead-coupled SOMAmers. Experiments were conducted in batches of 96 samples. After several washing steps, bead-bound proteins were biotinylated. Biotinylated target proteins complexed with fluorescence-labeled SOMAmers were photo cleaved. After recapture on streptavidin beads and additional washing steps, SOMAmers were eluted and quantified using customized arrays containing SOMAmer-complementary oligonucleotides. Based on standard samples that were included on each plate, the resulting raw intensities were processed in a data analysis work flow that accounted for hybridization normalization, median signal normalization and signal calibration. This protocol was implemented by SomaLogic Inc. (Boulder Colorado, USA) who received one-thousand blood samples from the KORA F4 study for analysis. From the resulting data, three samples were discarded, where one sample failed SOMAscan QC and two of the shipped samples were found to have been incorrectly pulled from the bio-bank, leaving a total of 997 samples. Of these 997 samples, 944 samples had both methylation and genotyping measurements and were used in this study. Data for 1129 SOMAmer probes (SOMAscan assay V3.2) was obtained for these samples. Five of the probes failed SOMAscan QC and one was not measured in QMDiab, leaving a total of 1123 probes for analysis.

### Genotyping (KORA)

The Affymetrix Axiom Array was used to genotype 3,788 samples of the KORA S4 of which 944 were used in this study. After thorough quality control (total genotyping rate in the remaining SNPs was 99.8%) and filtering for minor allele frequency (MAF) >1%, a total of 509,946 autosomal SNPs was used to impute more SNPs. Shapeit v2 was used to infer haplotypes from the available SNPs using the 1000G phase 3 haplotype build 37 genetic maps. Impute2 v2.3.2 was used for imputation. Variants with certainty <0.95, information metric <0.7, missing genotype (geno 0.2), Hardy–Weinberg equilibrium (hbe) exact test *p*-value < 1 × 10^−6^, or with MAF < 0.01 were all excluded. A total of 8,263,604 variants with a total genotyping rate of 0.97 were kept for the analysis.

### Phenotype/metabolic data (KORA)

We used measurements from a maximum of 1727 and 999 individuals overlapping the methylation and proteomics data respectively for the following phenotypes: hypertension (defined as self-reported hypertension diagnosis (>140/90 mmHg), or medically controlled known hypertension (as per ISH-WHO 1999)), myocardial infarction (self-reported), metabolic syndrome (MetS was defined according to the harmonized definition^69^ by the presence of three or more of the following criteria: (1) waist circumference ≥94 cm in men and ≥80 cm in women; (2) fasting serum triglycerides ≥150 mg/dl or drug treatment for elevated triglycerides (fibrates or niacin); (3) serum high density lipoprotein cholesterol (HDL) <40 mg/dl in men and <50 mg/dl in women or drug treatment for reduced HDL (fibrates or niacin); (4) systolic blood pressure ≥130 mmHg or diastolic blood pressure ≥85 mmHg or treatment with antihypertensive medication; (5) fasting serum glucose level ≥100 mg/dl or drug treatment of elevated glucose.), type 2 diabetes (defined as self-reported T2D diagnosis and validated using healthcare records and participants reported as non-diabetic were validated using OGT test), body mass index (defined as the ratio of weight in kilograms to the height in square meters), alcohol consumption in grams/day, total cholesterol, HDL, LDL, and triglycerides, to search for associations with our pQTMs. Using fasting samples, total cholesterol was measured using the cholesterol-esterase method (CHOL Flex, Dade-Behring, Germany), HDL and triglycerides were measured using the TGL Flex and AHDL Flex methods (Dade-Behring), respectively, and LDL was measured by the direct method (ALDL, Dade-Behring).

### Study population (QMDiab)

The Qatar Metabolomics Study on Diabetes (QMDiab) is a cross-sectional case-control study that was carried out in 2012 at the Dermatology Department in Hamad Medical Corporation (HMC Doha, Qatar). This study and comprises 388 study participants from Arab and Asian ethnicities^17^. The QMDiab study was approved by the Institutional Review Boards of HMC and WCM-Q under research protocol number 11131/11). All study participants provided written informed consent. A subset of 344 samples having joint methylation, proteomics, and genotyping data were used in this study.

### DNA methylation (QMDiab)

The Illumina Infinium HumanMethylation450 (450K) BeadChip array was used^70^ for genome-wide DNA methylation profiling of over 485,000 methylation sites. of 359 samples. All samples passed quality assessment of assay performance requirements implemented in the Genome Studio software integrated controls dashboard^15^. Normalization was performed using the Lumi:BMIQ pipeline, which includes color bias adjustment, quantile normalization (QN), and beta mixture quantile dilation normalization (BMIQ). 344 of the 359 methylation samples overlapped with both the proteomics and genotyping data and were used in this study.

### Proteomics (QMDiab)

The SOMAscan platform of the WCM-Q proteomics core was used to quantify a total of 1129 protein measurements in originally 356 plasma samples from QMDiab^2^. Protocols and instrumentation were provided and certified following SomaLogic Inc. standards and requirements. Qualified SomaLogic personnel supervised the experiments on-site. No samples or probe data were excluded. 344 of the 356 samples overlapped with both the methylation and genotyping data and were used in this study.

### Genotyping (QMDiab)

Genotyping was carried out using the Infinium Human Omni 2.5-8 V1.2 Beadchip array for originally 353 samples^2^. After stringent quality control, 1,221,345 variants were used impute a total of 18,829,416 variants that are used in this study. In total, 344 of the 353 samples that overlapped with both the methylation and proteomics data were used in this study.

### Metabolomics (QMDiab)

A total of 2251 metabolomics measurements from QMDiab samples were obtained^15^. The semi-quantitative non-targeted UPLC-MS/MS and GC-MS platform from Metabolon Inc. was used, yielding measurements of metabolic traits (758 from plasma, 891 from urine, and 602 from saliva). Briefly, non-targeted metabolic profiling at Metabolon was achieved in 330 saliva, 358 in blood plasma, and 360 urine QMDiab samples using ultrahigh-performance liquid-phase chromatography and gas chromatography separation, coupled with tandem MS using established procedures^71^.

### Statistical analysis

The CpG methylation *b*-values were transformed to *M*-values, which are considered more statistically valid for association analysis^72^. M-values are defined as the log2 ratio of the intensities of methylated versus un-methylated probe. The protein measurements were log scaled. The transformed methylation and protein data were then winsorized, that is, outliers that fall in the <5th and the above 95th quantiles were replaced by the respective quantile values. The CpG and protein values were then standardized (*z*-scored) to a mean equal to zero and a standard deviation of one. A series of step-wise protein-epigenome-wide association studies (pEWASs) were carried out, where a set of covariates were iteratively regressed out from the proteomic and methylation measures separately using linear models. Residuals of the proteomic and methylation measurements were computed after regressing out covariates in the following order: sex, white blood cell coefficients, cis-SNPs, age, smoking, body mass index, and diabetes. A pEWAS having the residual DNA methylation (M-values) as the dependent variable and the residual log-scaled protein as the independent variable was then carried out at each step. The multiple-testing Bonferroni corrected level of significance for methylation sites (*N* = 470,837) and proteins (*N* = 1123) was *p*_CpG~Protein_ <9 × 10^−11^ (0.05/1123/470,837).

The same preprocessing of data and statistical analysis procedure of the pEWAS was carried out for the replication in QMDiab as done in KORA with one minor variation: In QMDiab, the first three principal components (PCs) of the genotyping data (genoPC1, genoPC2, and genoPC3) along with the first three principal components of the proteomics data (somaPC1, somaPC2, and somaPC3) were added as covariates in addition to the sex in that step of the analysis. These PCs are considered as standard co-variates of the QMDiab study^2^. The genetic PCs account for the ethnic variability of the QMDiab cohort and the proteomics PCs account for a moderate level of observed cell lysis. In KORA, possible effects from population stratification have already been excluded in previous studies^3^. Therefore, no adjustment for population structure was performed in KORA. Furthermore, we computed the number of discovery pQTMs that had 95% replication power (determined by sampling) and the percentage of them having consistent directionality. We report the fraction of Bonferroni replicated pQTMs at 95% replication power and the fraction of nominally significant pQTMs with consistent directionality within all nominally significant pQTMs for all steps (Supplementary Data 3).

Upon visual inspection of the CpG versus protein scatterplots we excluded three associations at a single locus, as they appeared to be driven by an underlying SNP which had not been genotyped or imputed (confirmed by the fact that the assumed SNP allele distribution respected Hardy–Weinberg equilibrium). Already at the discovery stage we attempted to regress out any remaining contributions of large-scale variance that might have been captured by the principal components of the final CpG and protein levels, but did not find any.

The CpG/protein associations with KORA phenotypes/metabolites (hypertension, myocardial infarction, metabolic syndrome, type 2 diabetes, body mass index, alcohol consumption, total cholesterol, HDL, LDL, and triglycerides) were carried out using the log transformed, winsorized, standardized protein measurements and the winsorized, standardized *M*-values for the CpG measurements while adjusting for age and sex as covariates. The multiple-testing Bonferroni corrected level of significance was set at *p*_CpG~Phenotype_ < 5.6 × 10^−4^ (0.05/89) and *p*_Protein~Phenotype_ < 3.3 × 10^−3^ (0.05/15).

The metabolomics associations with CpG sites in QMDiab were computed using linear models and using the following covariates: age, sex, white blood cell coefficients, and genetic PCs. The multiple-testing Bonferroni corrected level of significance was set at *p*_CpG~Metabolite_ < 2.2 × 10^−5^ (0.05/2251/89).

### Cross reactivity of CpG markers and aptamers

To ensure that none of the remaining CpG-protein associations were mis-interpreted due to binding issues, we checked all involved CpG sites and proteins for cross reactivity. In methylation, cross-reactive probes are probes that bind at non-target sequence due to similarity in sequence. Additionally, several probes target polymorphic CpGs that overlap with SNPs. A list of cross-reactive or polymorphic CpGs in the Illumina Human Methylation 450K array are provided by Chen et al.^73^ and Price et al.^74^. A list of cross-reactive proteins was obtained from the study of Sun et al.^58^, who tested a subset of the Somalogic aptamers (SOMAmers) for cross-reactivity with homologous proteins that have at least 40% sequence similarity. In addition, we assessed the specificity of the SOMAlogic assay for the proteins in our final list of 98 associations using data provided by Emilsson et al.^59^, where direct assessment of aptamer specificity using data dependent analysis and multiple reaction monitoring mass spectrometry after SOMAmer enrichment in biological matrices. Two of our final proteins (B2M and FCGR3B) were directly validated via mass spectrometry in blood plasma, two proteins (CD163 and SIGLEC14) in blood serum, and four proteins (C4A, CLEC11A, LAG3, and PRTN3) in other biological matrices. Additionally, validation is inferred for the remaining seven proteins, thus providing further assurance regarding the issue of cross-reactivity.

### Enrichment of pQTMs for various CpG characteristics

Enrichment analysis for the various characteristics of CpG sites was based on the Illumina 450K manifest. Enrichment/depletion was computed for various UCSC gene regions describing the CpG position (TSS200, TSS1500, 5′UTR, Body, and 3′UTR), the location of the CpG relative to the CpG island (shore, shelf, upstream of the CpG island, and downstream of the CpG island), regulatory features as determined by the ENCODE consortium (promotor, gene, non-gene, unclassified, and those features also being cell type specific), and other features including differentially methylated regions (DMR), cancer-specific differentially methylated regions (CDMR), Enhancer, and DNase I Hypersensitivity sites (DHS). A Fisher exact test for count data (fisher.test in R) was used to compute the odds ratio, the 95% confidence interval for the odds ratio, and *p*-value. An odds ratio greater than one represented enrichment, and the contrary, depletion.

Finally, we crossed our pQTMs against published Hi-C data for both inter-chromosomal and intra-chromosomal contact^19^ at 1 kb base pair delimited resolution and quality threshold of E30 in the GM12878 LCL. Both the CpG and protein were put in the relevant 1 kb block, and for those blocks, the chromosomal contact value was retrieved from the Rao et al. data^19^. If a Hi-C contact was indicated between a CpG and protein, we flagged the pQTM as positive for Hi-C contacts. As background, we used randomized pQTM combinations matching in size to those in the actual analysis.

### Association between CpG methylation and gene expression

We used the BIOS QTL browser (http://genenetwork.nl/biosqtlbrowser, accessed 28 September 2018)^5^ to identify cis-methylation eQTMs (correlations between gene expression and CpG methylation that are located in cis). eQTMs were in a window of 250 kb around the TSS of the transcript. This eQTM data was generated using RNA-seq data for 2101 of 3841 Dutch individuals. 12,809 unique CpG sites correlated with 2842 unique genes in cis CpG-level at a false discovery rate FDR <0.05.

### Pathway analysis

The networks were generated through the use of IPA (QIAGEN Inc., http://www.qiagenbioinformatics.com/products/ingenuity-pathway-analysis)^18^. We searched for connections between genes located near the identified CpG sites and the target genes of the associated proteins (accessed September 2018, database version 44691306). We used the path explorer tool to identify direct or indirect relationships using the ingenuity knowledge base by selecting the most stringent available confidence level (experimentally observed) only.

### Reporting summary

Further information on research design is available in the Nature Research Reporting Summary linked to this article.

## Supplementary information

Supplementary Information

Supplementary Data 1

Supplementary Data 2

Supplementary Data 3

Supplementary Data 4

Supplementary Data 5

Supplementary Data 6

Supplementary Data 7

Supplementary Data 8

Supplementary Data 9

Supplementary Data 10

Supplementary Data 11

Supplementary Data 12

Description of Additional Supplementary Files

Reporting Summary

## Data Availability

Complete summary statistics and association data across all measured CpG probes and proteins in the discovery cohort are available for download at: https://wcmq.box.com/s/f85fxv9ldvsshd1b1d0qbfwde75no1x4. The informed consent given by the study participants does not cover posting of participant level phenotype and genotype data in public databases. However, data are available upon request from KORA-gen (http://epi.helmholtz-muenchen.de/kora-gen). Requests are submitted online and are subject to approval by the KORA board.
