# Supplementary material for: Epigenetics meets proteomics in an epigenome-wide association study with circulating blood plasma protein traits
Source: Nat Commun. 2020 Jan 3;11:15. doi: 10.1038/s41467-019-13831-w (PMC6941977; doi:10.1038/s41467-019-13831-w)
Supplement: Supplementary file 1 — Supplementary Information [file 41467_2019_13831_MOESM1_ESM.pdf]

Supplementary Information

Epigenetics meets proteomics in an epigenome-wide association study with circulating blood plasma protein traits

Zaghlool et al.

Contents:

Supplementary Figure 1: Replication in the QMDiab study.

Supplementary Figure 2: Manhattan plots for step-wise pEWAS (Figures 2a-2h). **Supplementary Figure 3**: Step-wise inflation per protein (histograms).

Supplementary Figure 4: Enrichment/depletion barplots for various CpG designations.

Supplementary Figure 5: Association between *NLRC5* methylation (cg07839457) and the urinary 1,3,7,-trimehylurate and urinary neopterin (QMDiab).

Supplementary Figure 6: Correlation plot of 72 CpG sites associated with pappalysin-1 (PAPPA).

Supplementary Figure 7: Correlation plot of CpG sites and proteins (pQTMs) in the NLRC5 network.

Supplementary Note 1: Replication of previous pEWAS**.**

Supplementary Note 2: Details of pQTMs eliminated at each step of the pEWAS. **Supplementary Note 3:** Details for all connections in the PAPPA network**.**

Supplementary Note 4: Details for all connections in the NLRC5 network.

Supplementary Figure 1: Replication in the QMDiab study. We attempted replication of all pQTMs from the discovery study in QMDiab. Before regressing out any covariates, 12,606 of the 38,492 initially identified pQTMs replicated at a Bonferroni significance level (P < 0.05 / 38,492). We show how the number of replicated pQTMs varies at each subsequent regression step. The numbers next to the covariates represents the total number of Bonferroni significant pQTMs for that step. The list of replicated pQTMs identified at the different steps of the EWAS are provided in Supplementary Data 2. Vertical alignment represents pQTMs that were already significant in a previous step.

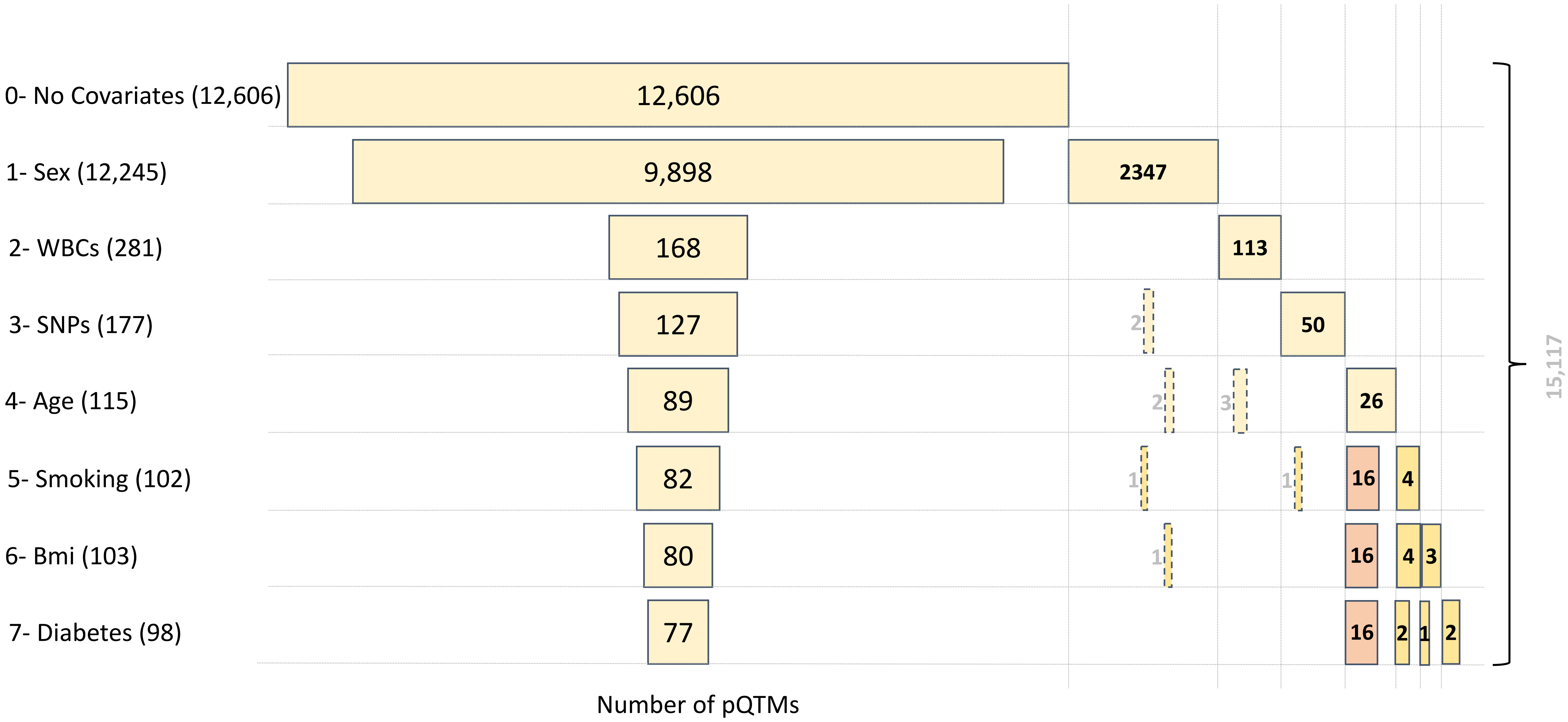

Supplementary Figure 2a: Manhattan plots for step-wise pEWAS (Step 0)

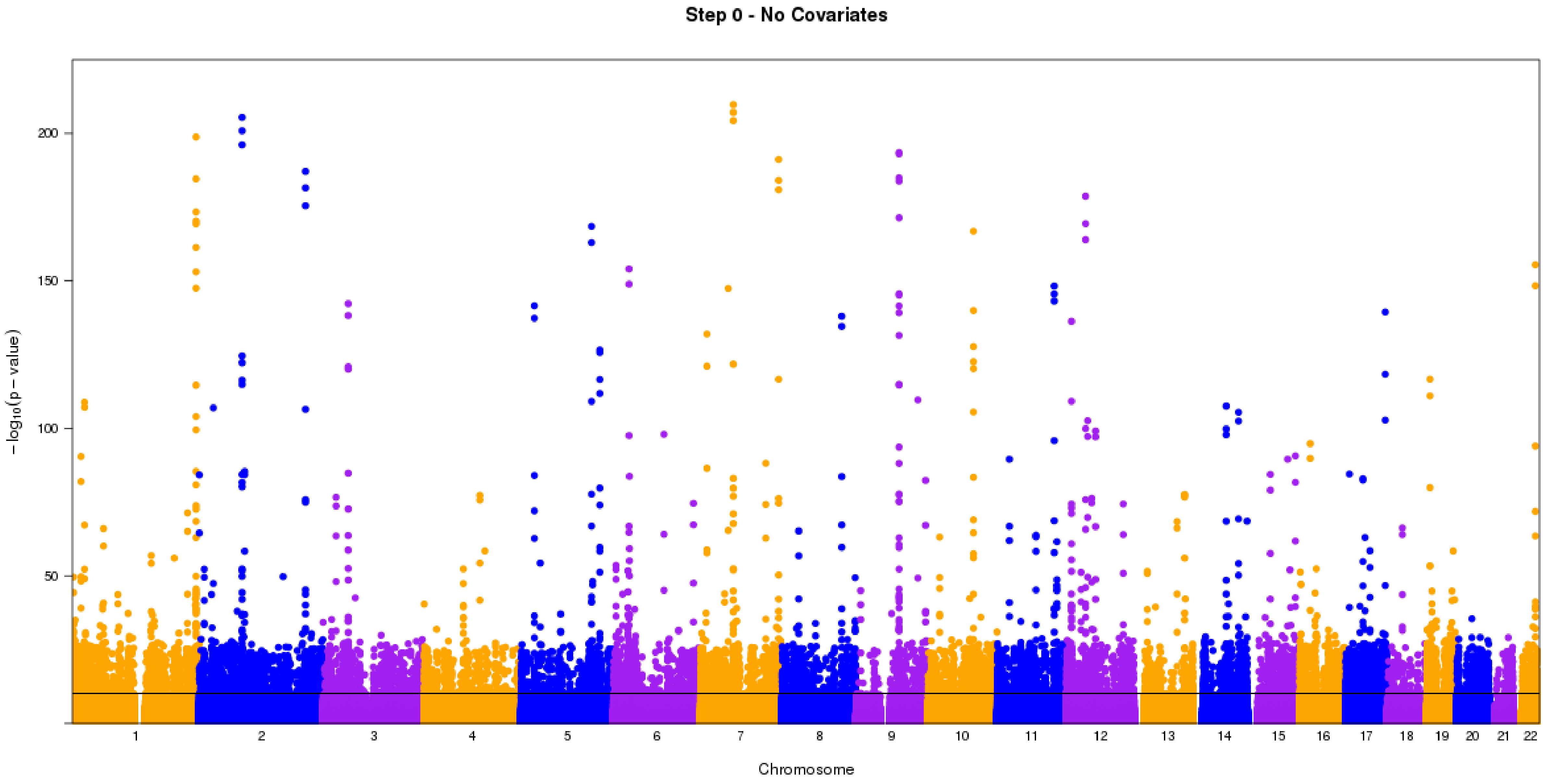

Supplementary Figure 2b: Manhattan plots for step-wise pEWAS (Step 1)

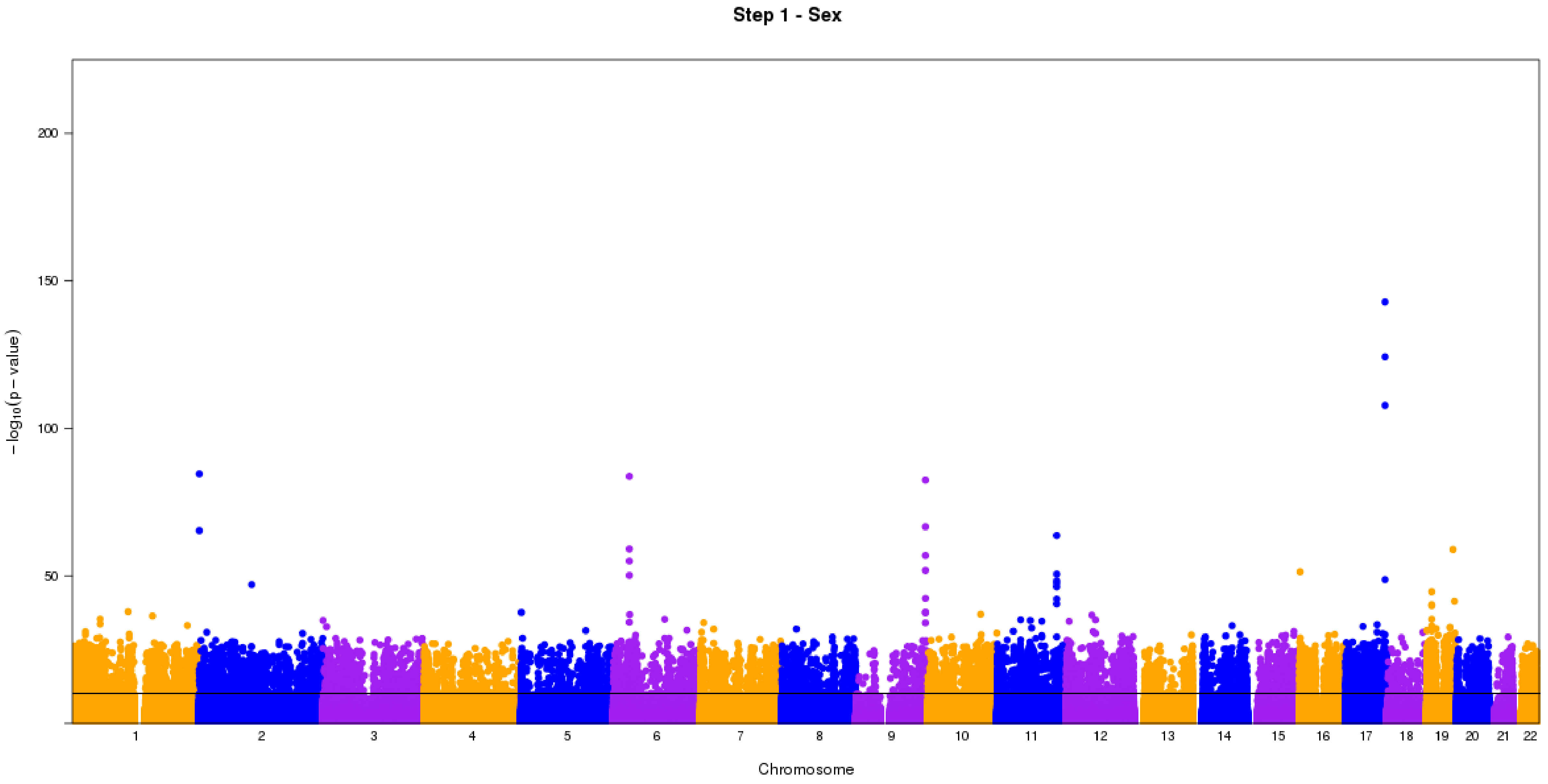

Supplementary Figure 2c: Manhattan plots for step-wise pEWAS (Step 2)

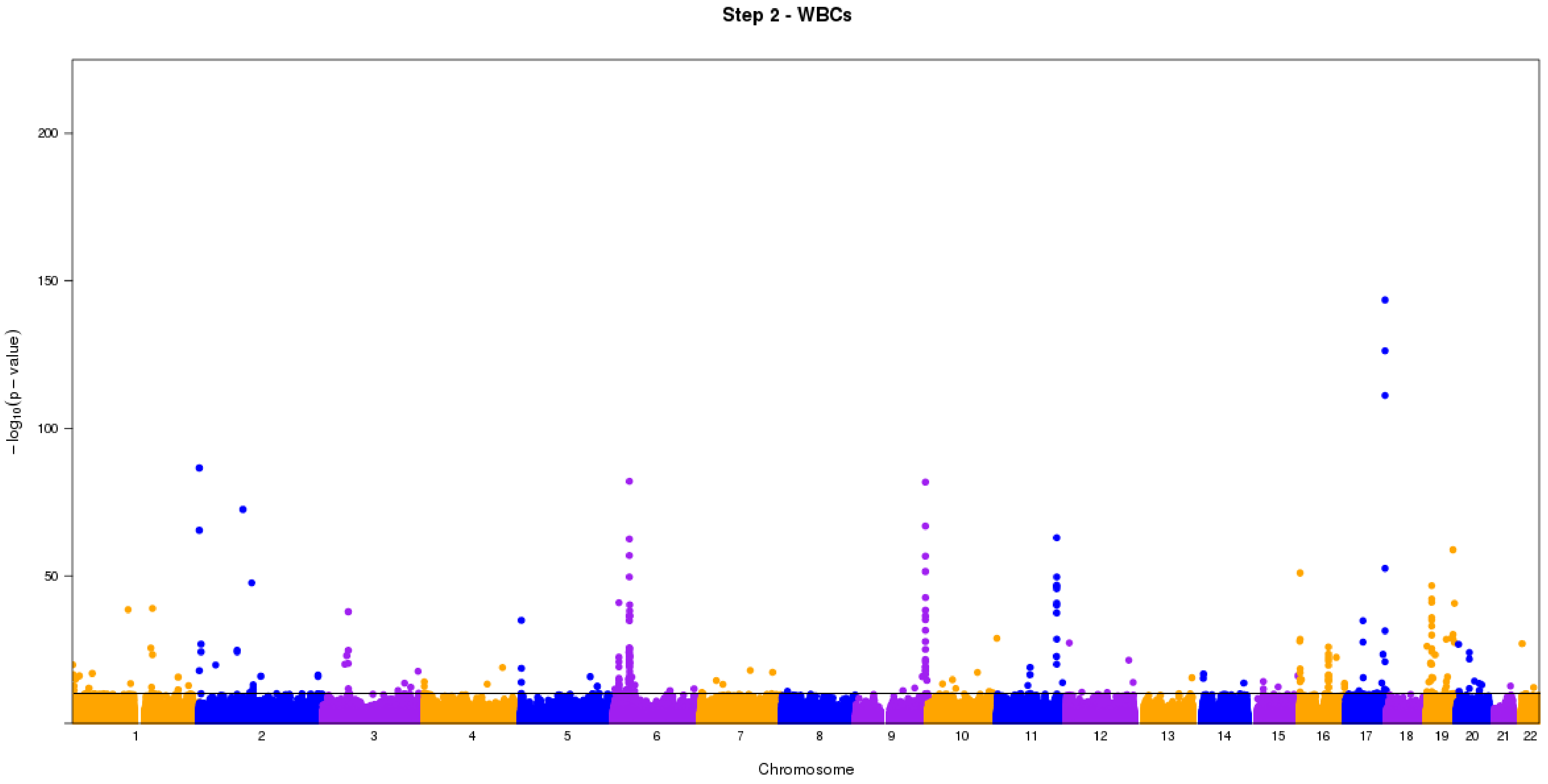

Supplementary Figure 2d: Manhattan plots for step-wise pEWAS (Step 3)

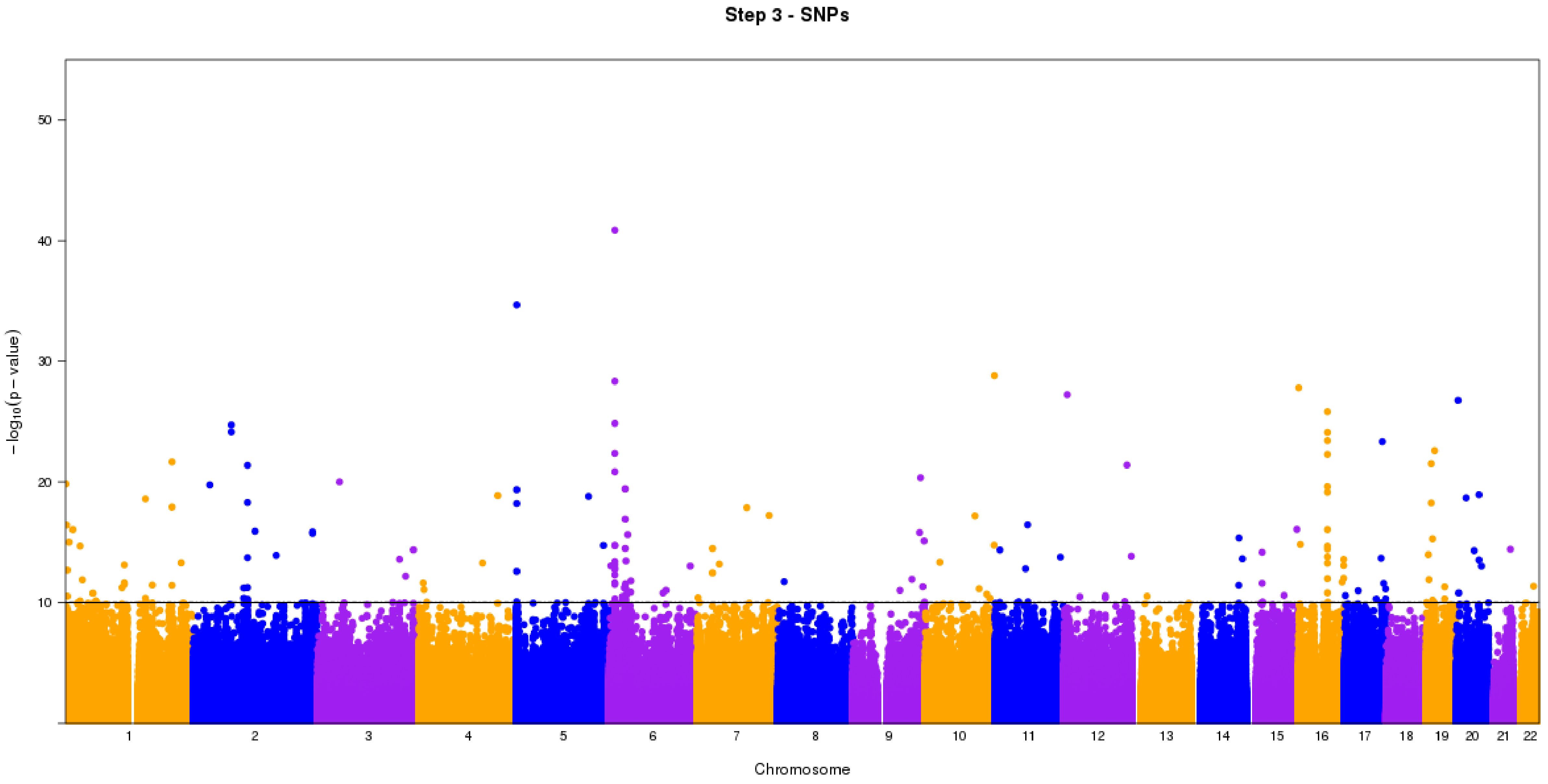

Supplementary Figure 2e: Manhattan plots for step-wise pEWAS (Step 4)

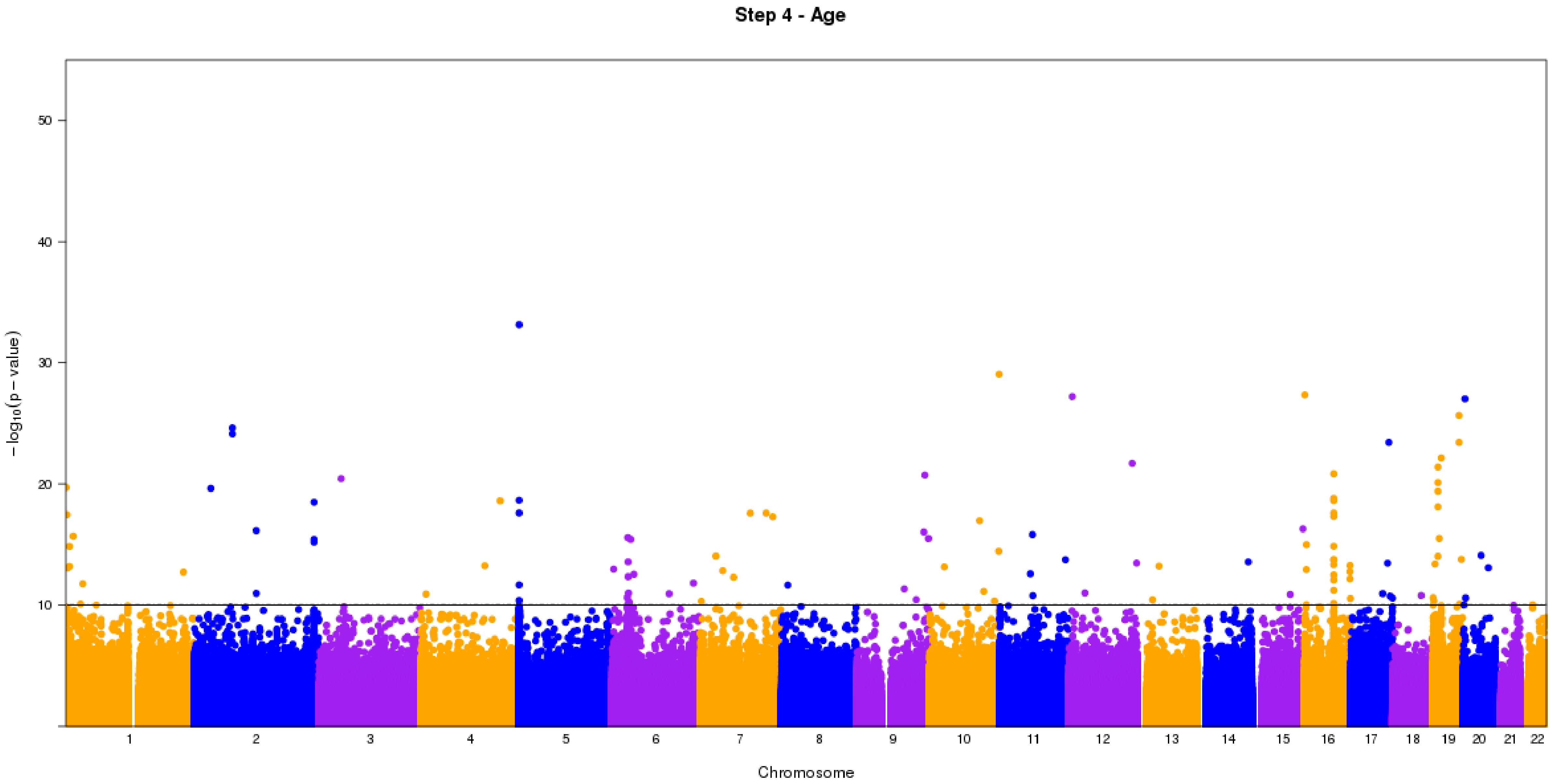

Supplementary Figure 2f: Manhattan plots for step-wise pEWAS (Step 5)

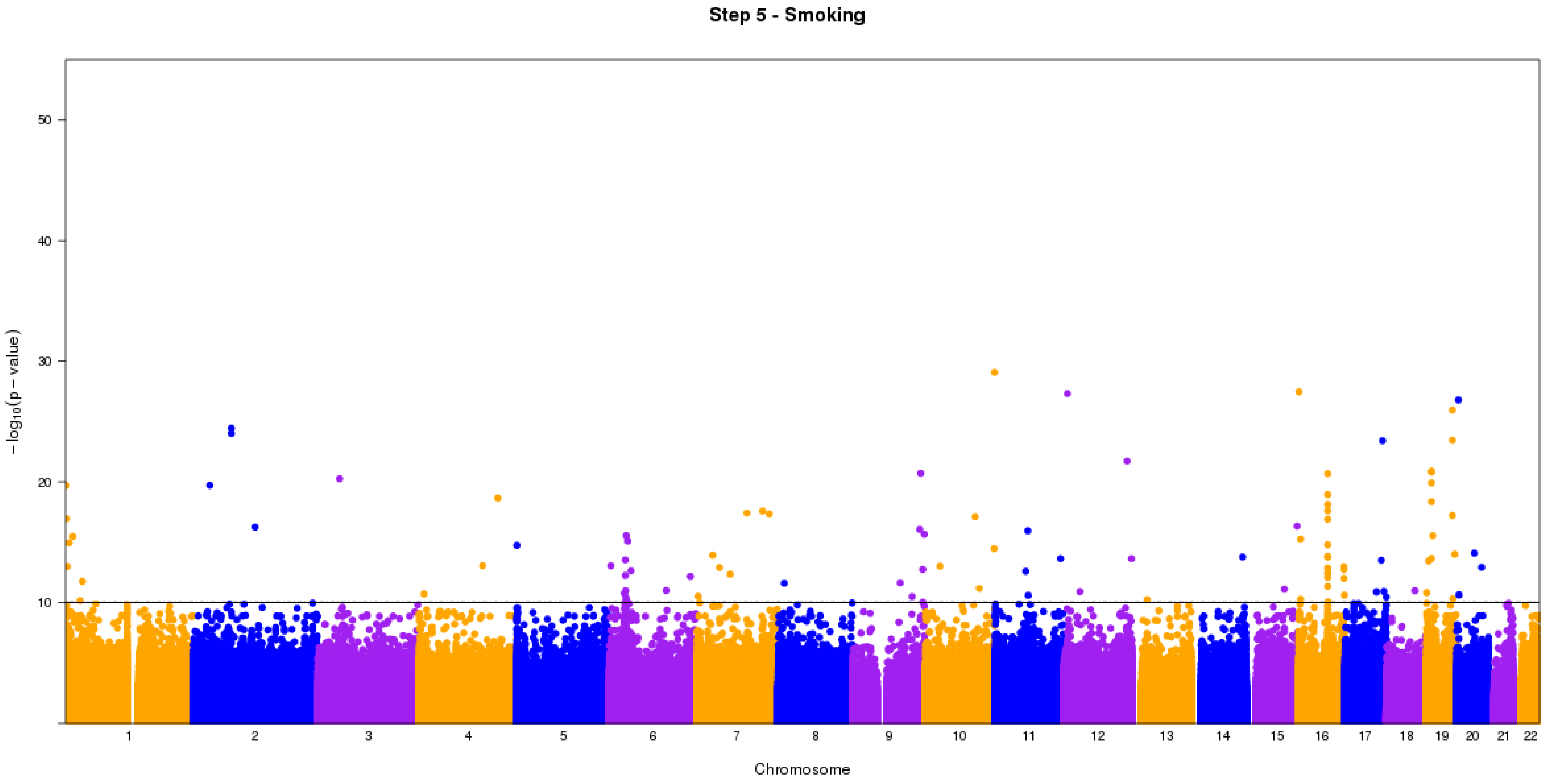

Supplementary Figure 2g: Manhattan plots for step-wise pEWAS (Step 6)

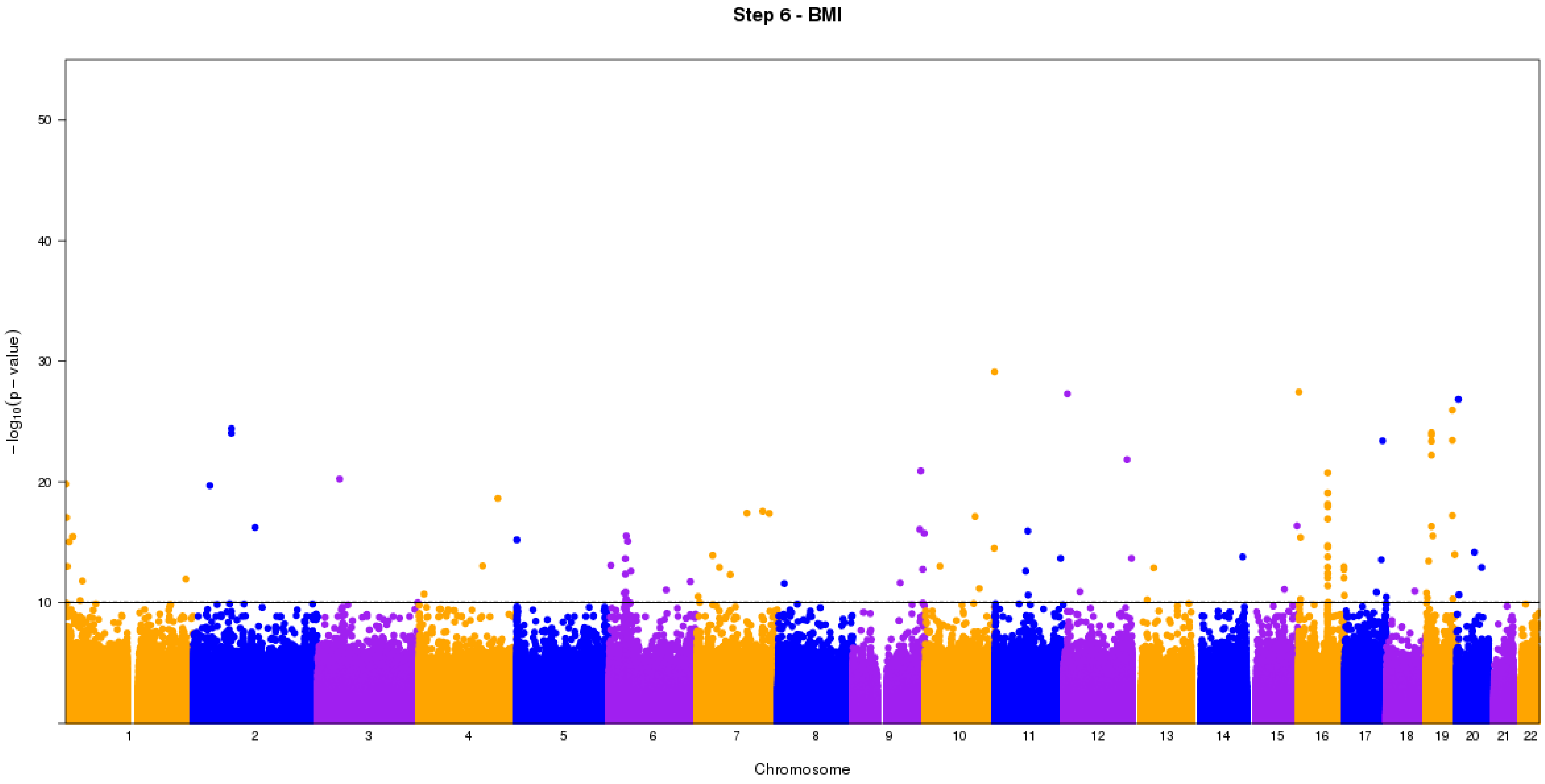

Supplementary Figure 2h: Manhattan plots for step-wise pEWAS (Step 7)

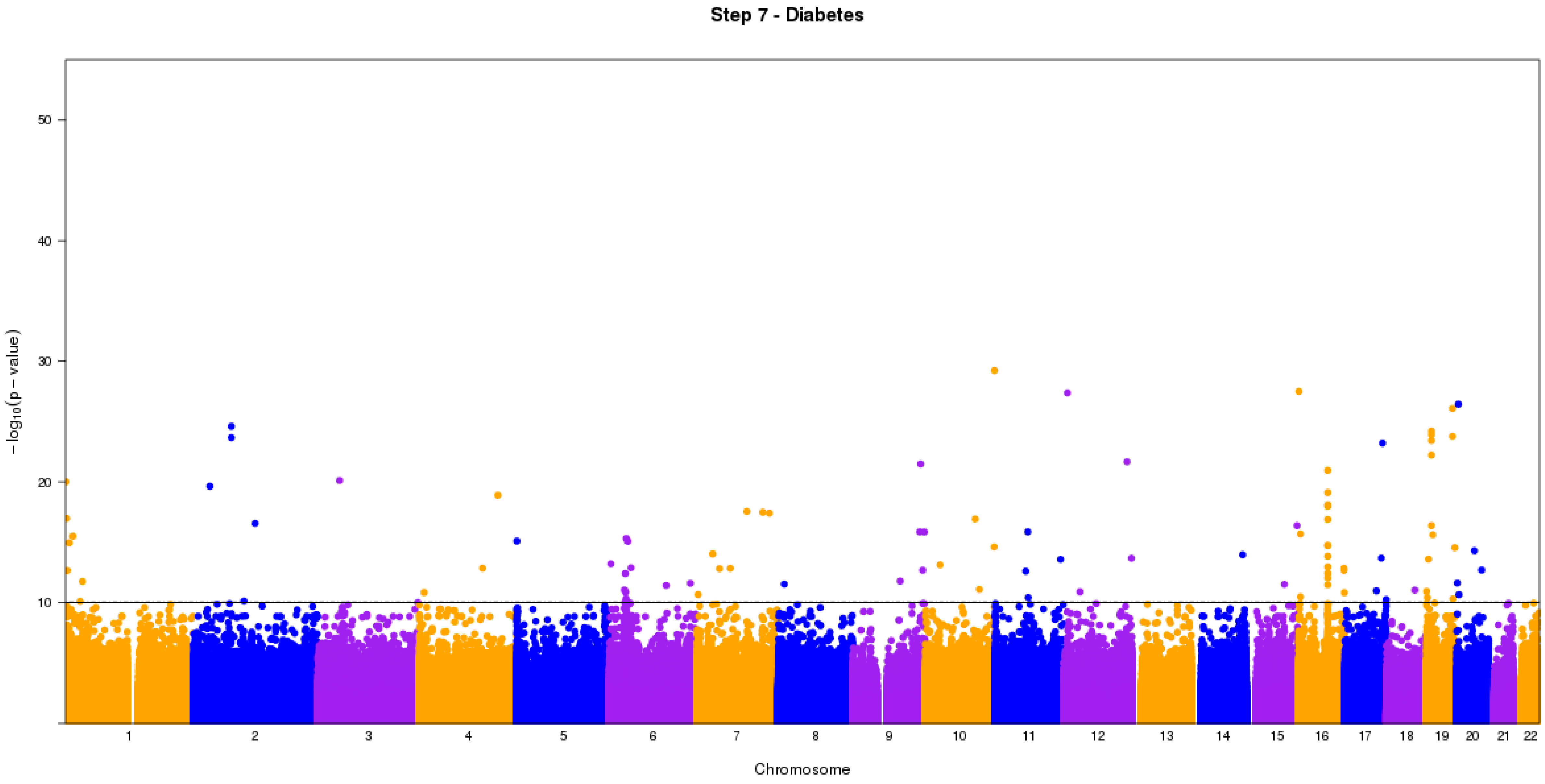

Supplementary Figure 3a: Step-wise inflation per protein (Step 0)

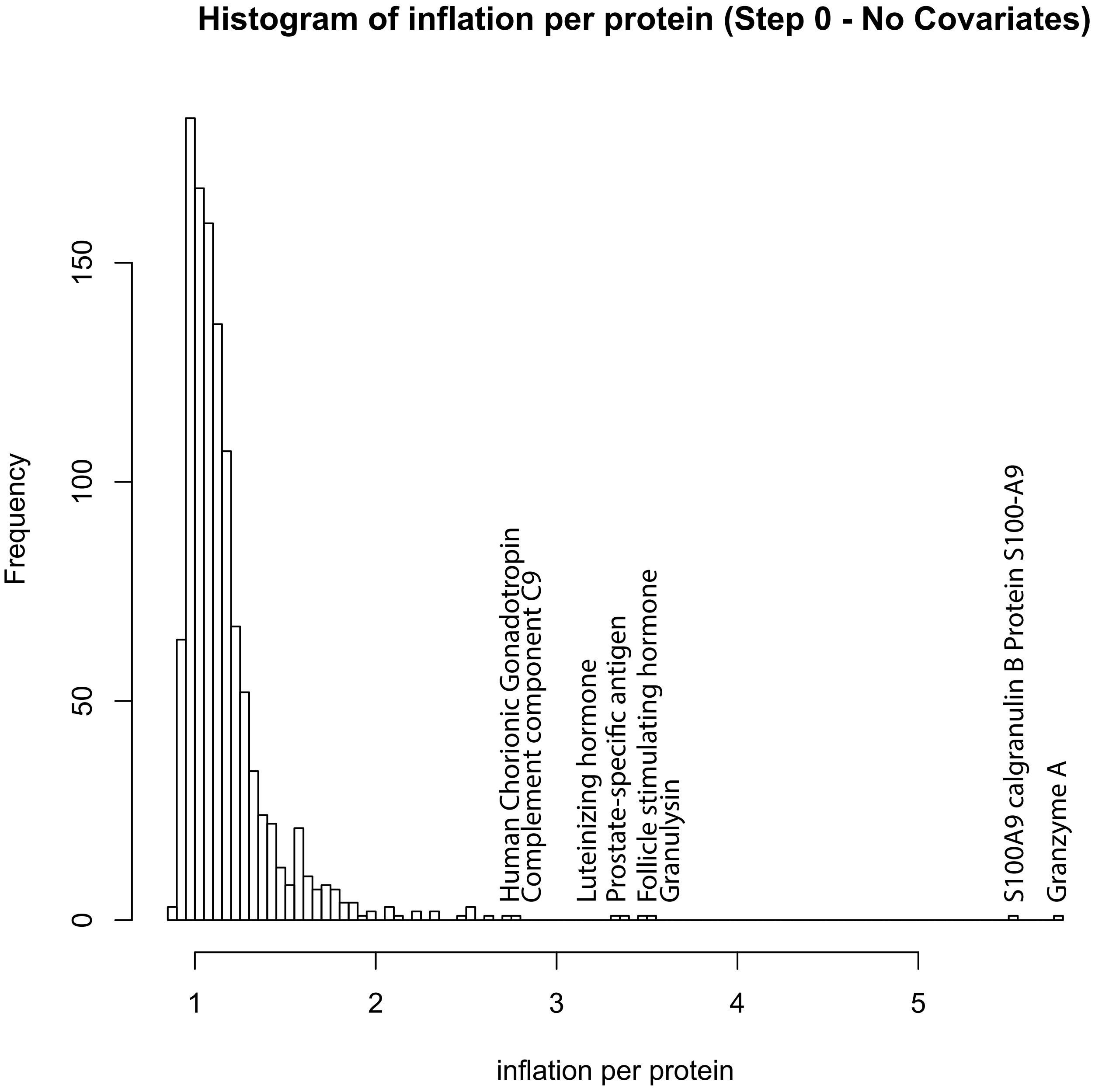

Supplementary Figure 3b: Step-wise inflation per protein (Step 1)

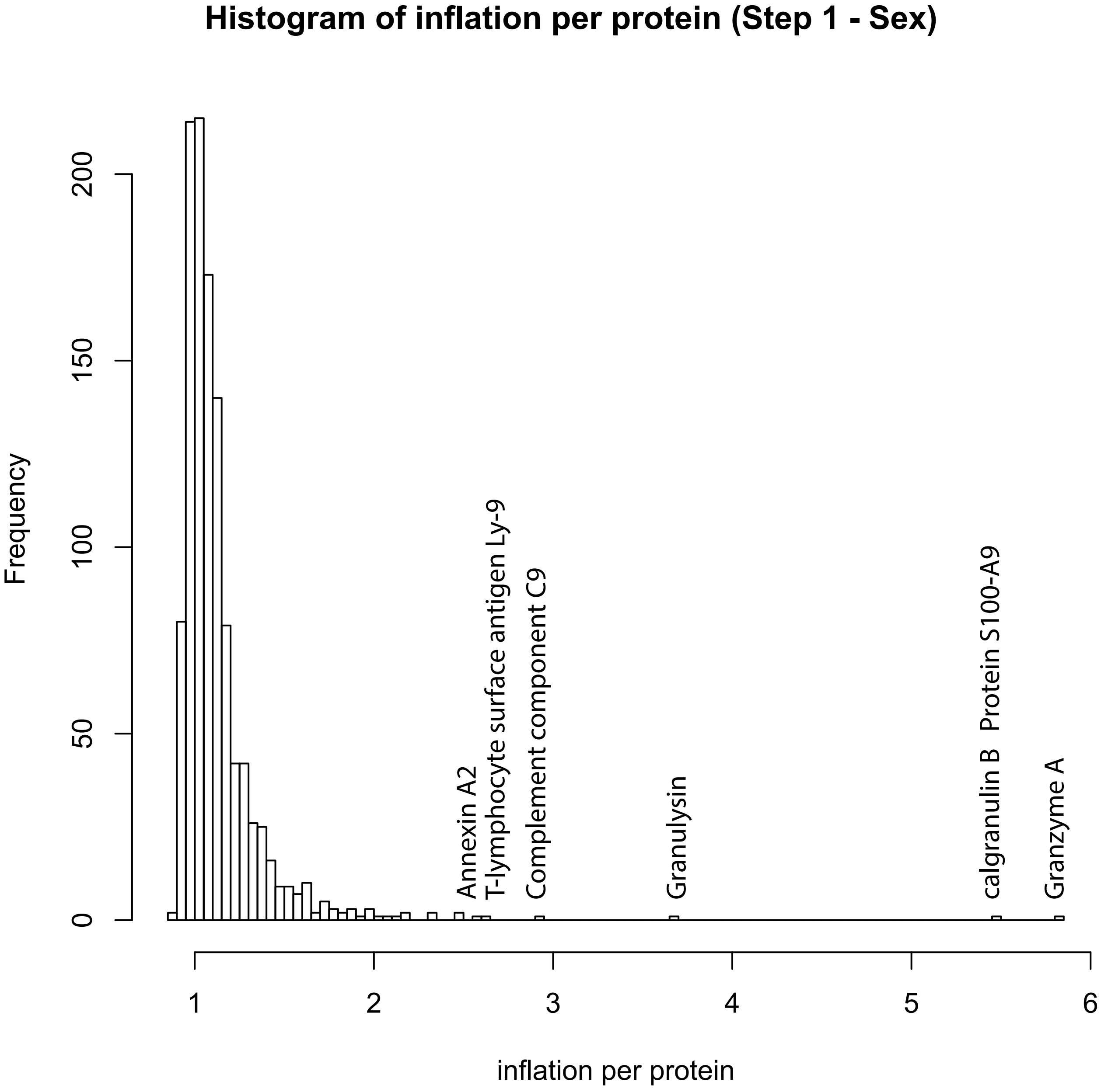

Supplementary Figure 3c: Step-wise inflation per protein (Step 2)

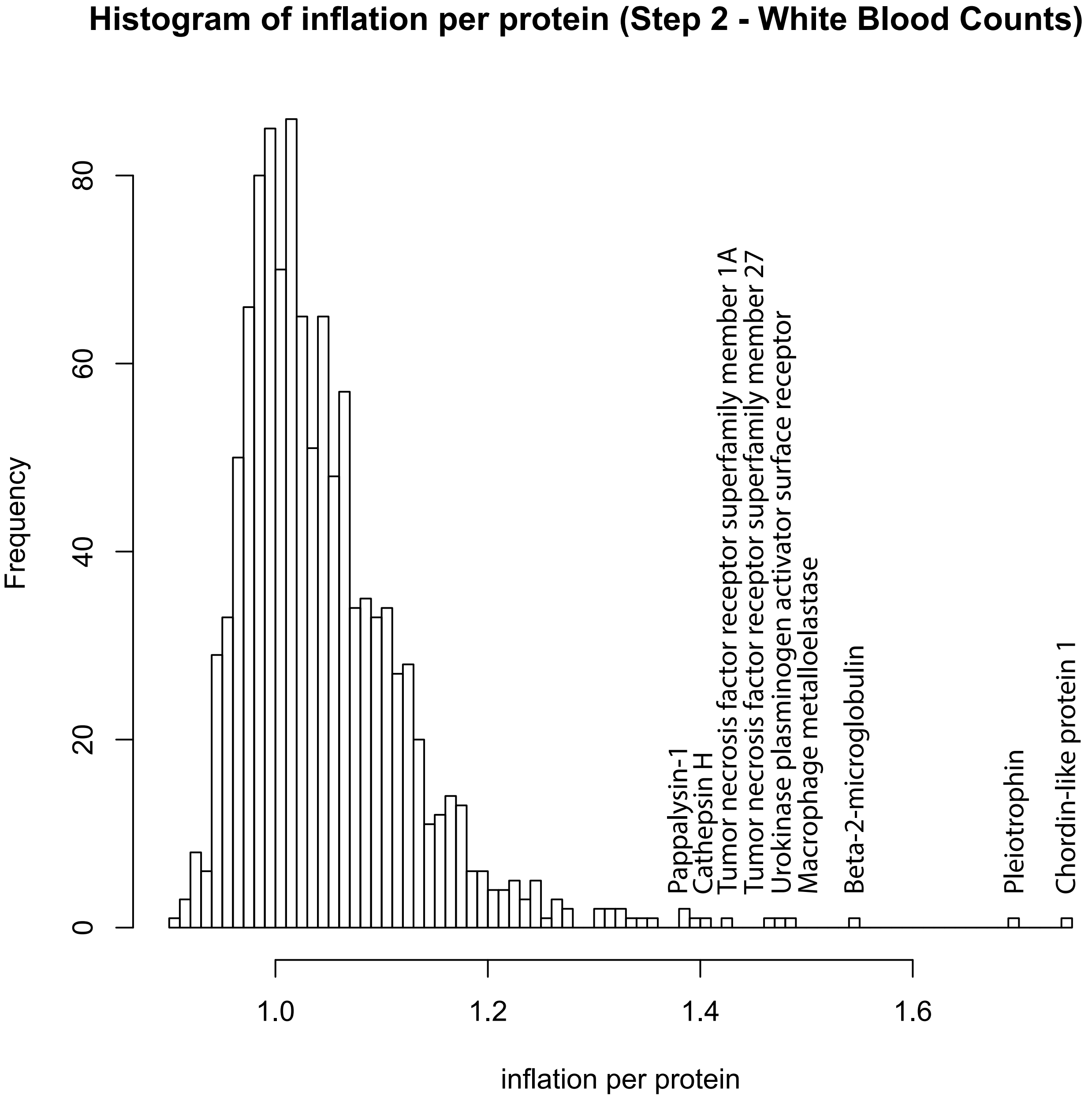

Supplementary Figure 3d: Step-wise inflation per protein (Step 3)

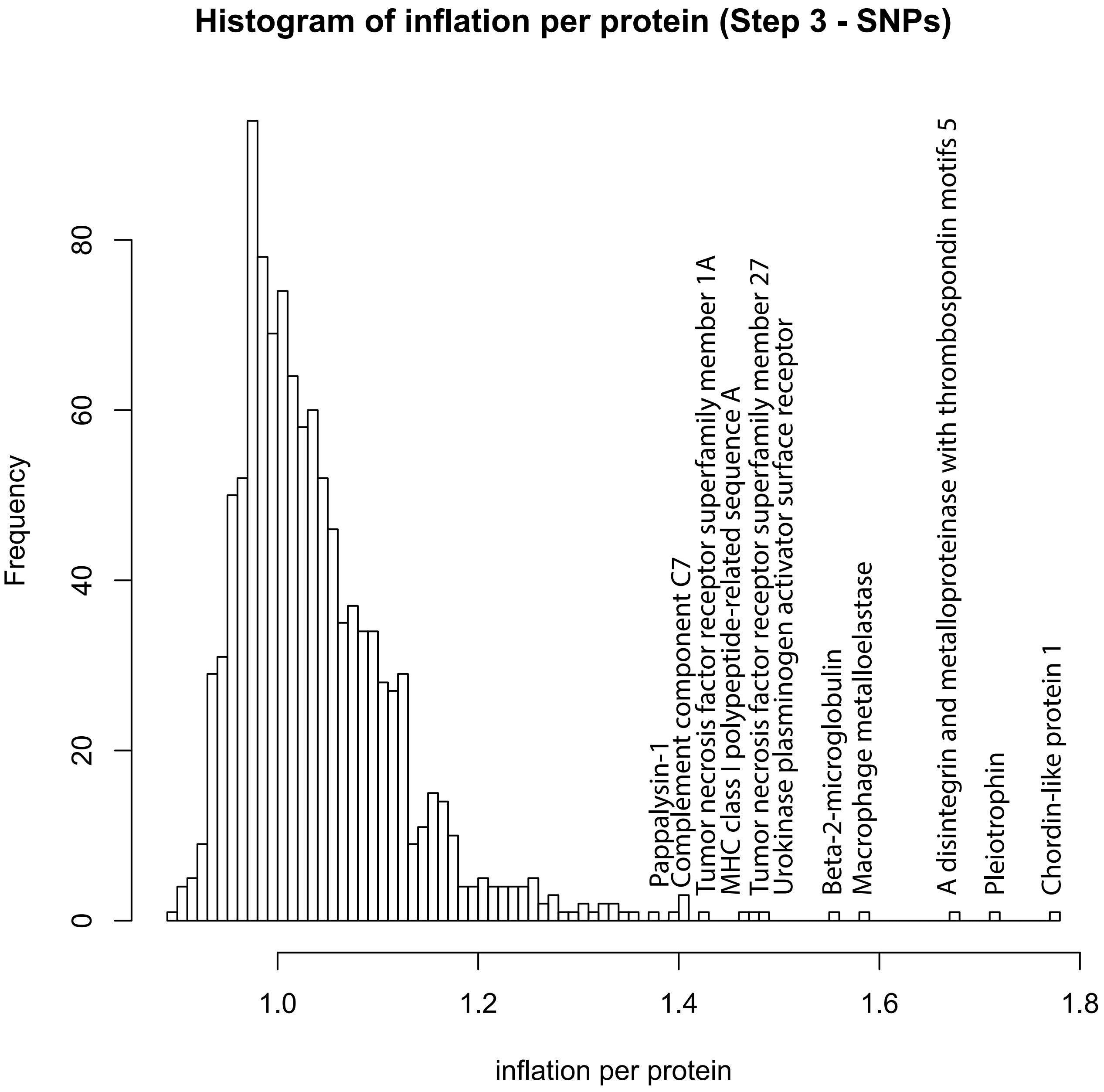

Supplementary Figure 3e: Step-wise inflation per protein (Step 4)

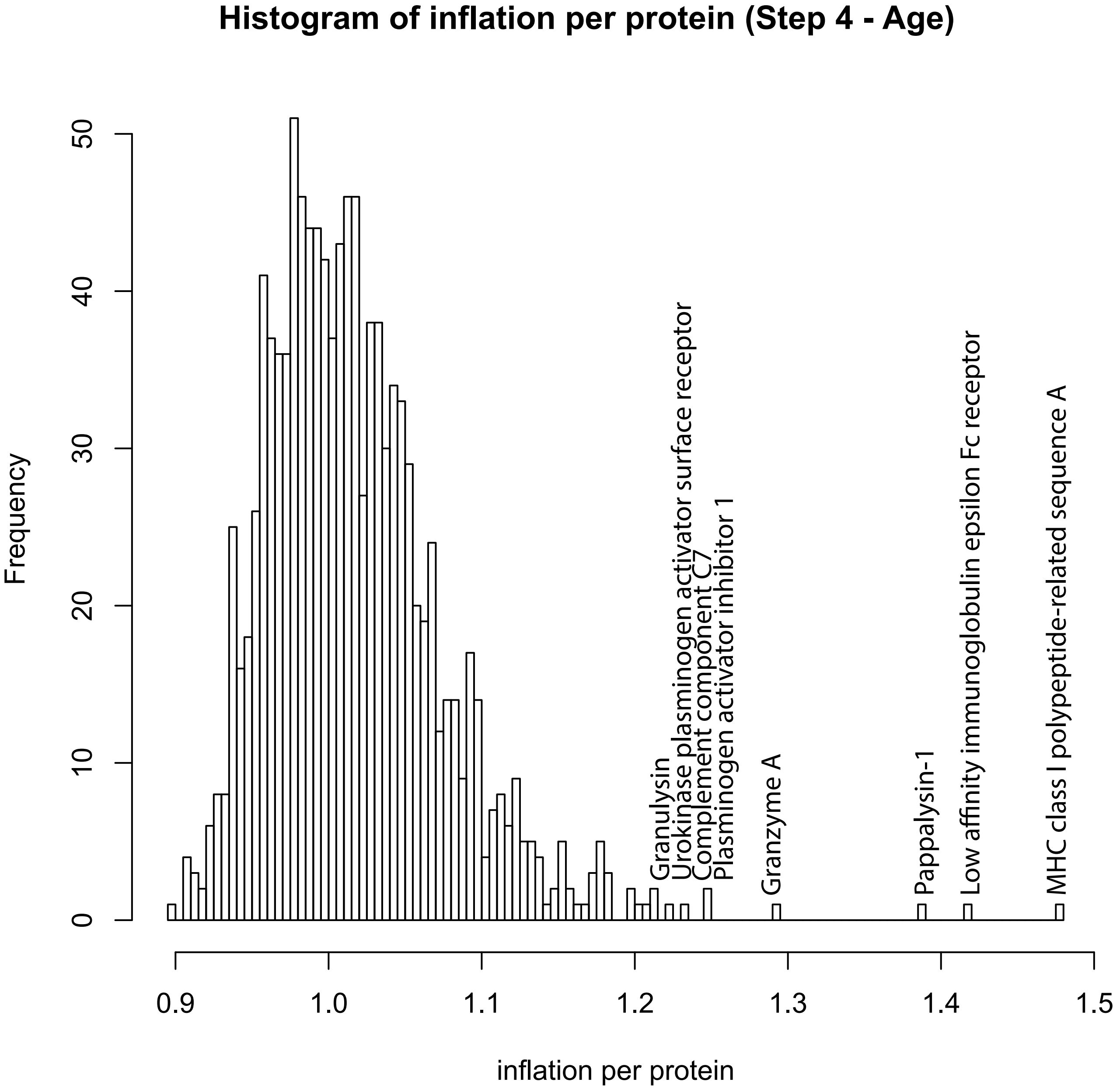

Supplementary Figure 3f: Step-wise inflation per protein (Step 5)

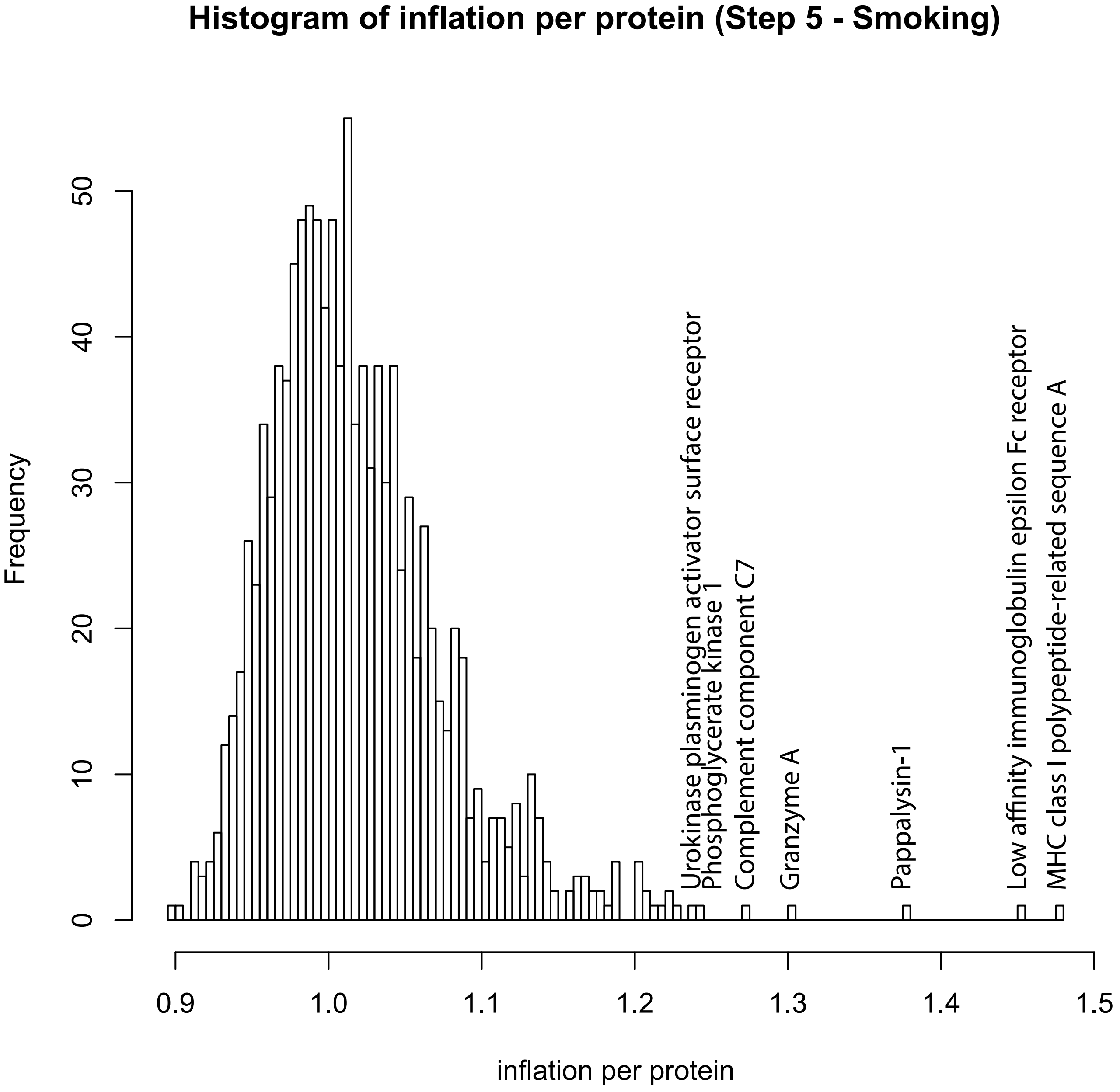

Supplementary Figure 3g: Step-wise inflation per protein (Step 6)

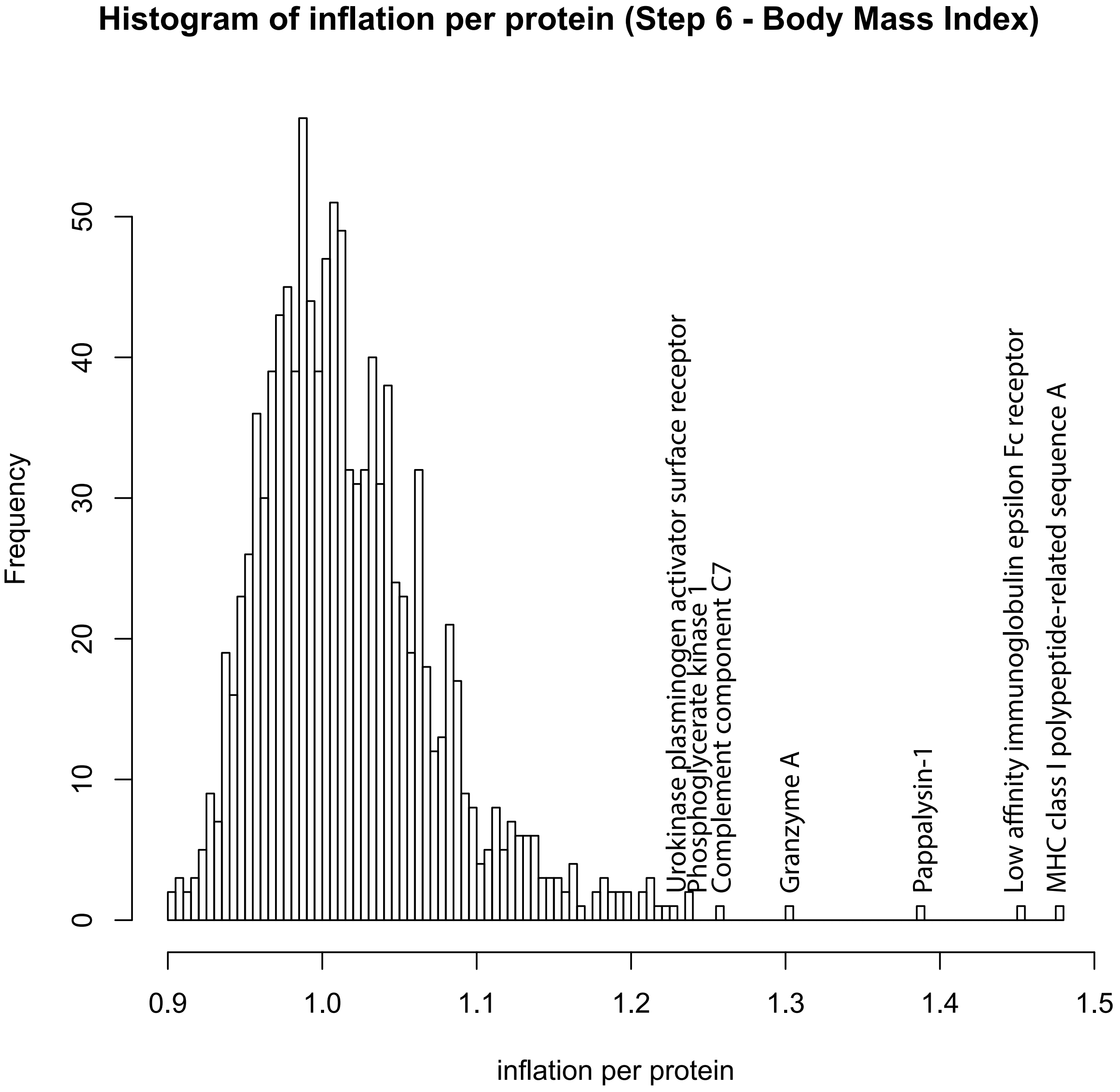

Supplementary Figure 3h: Step-wise inflation per protein (Step 7)

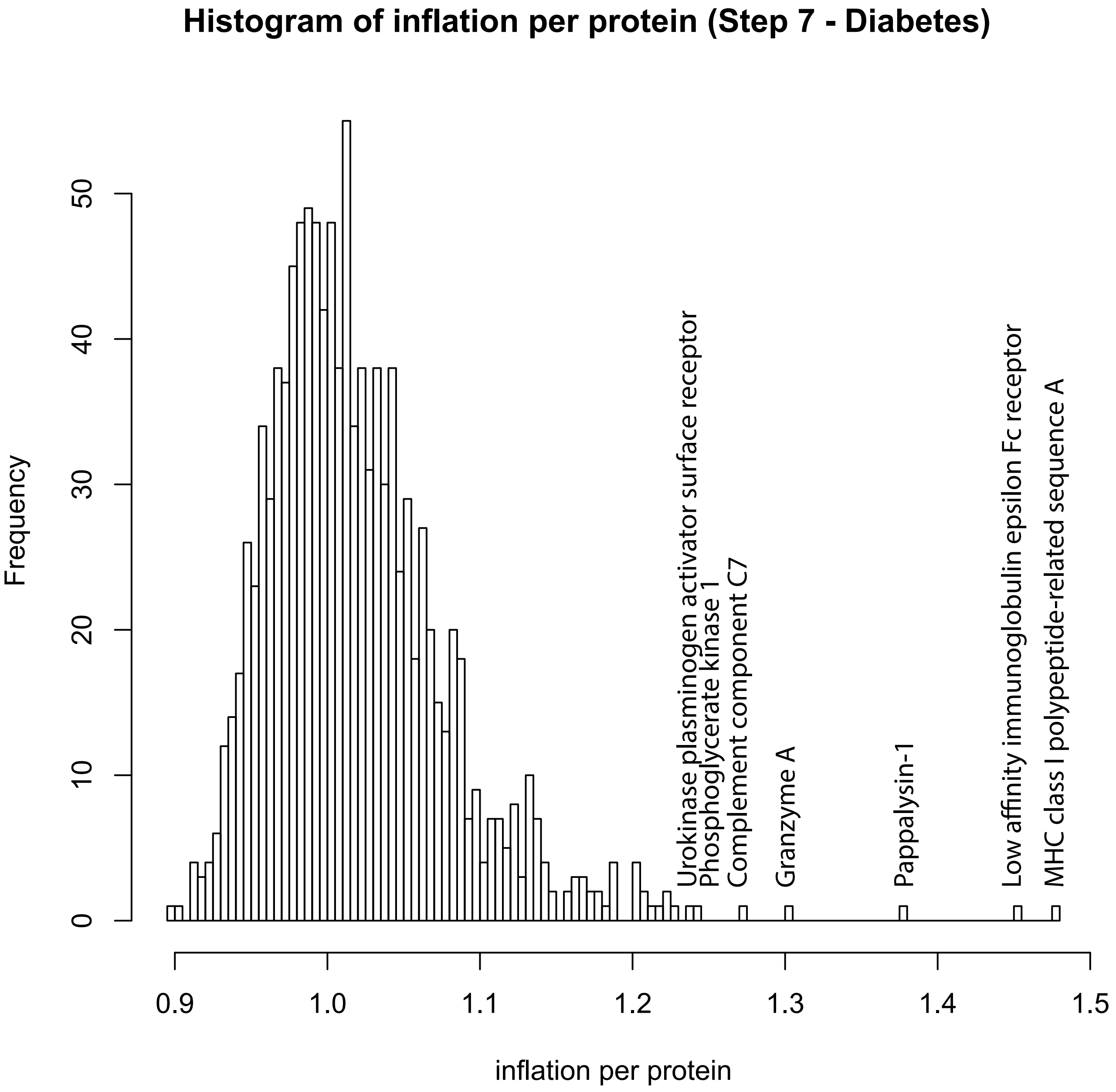

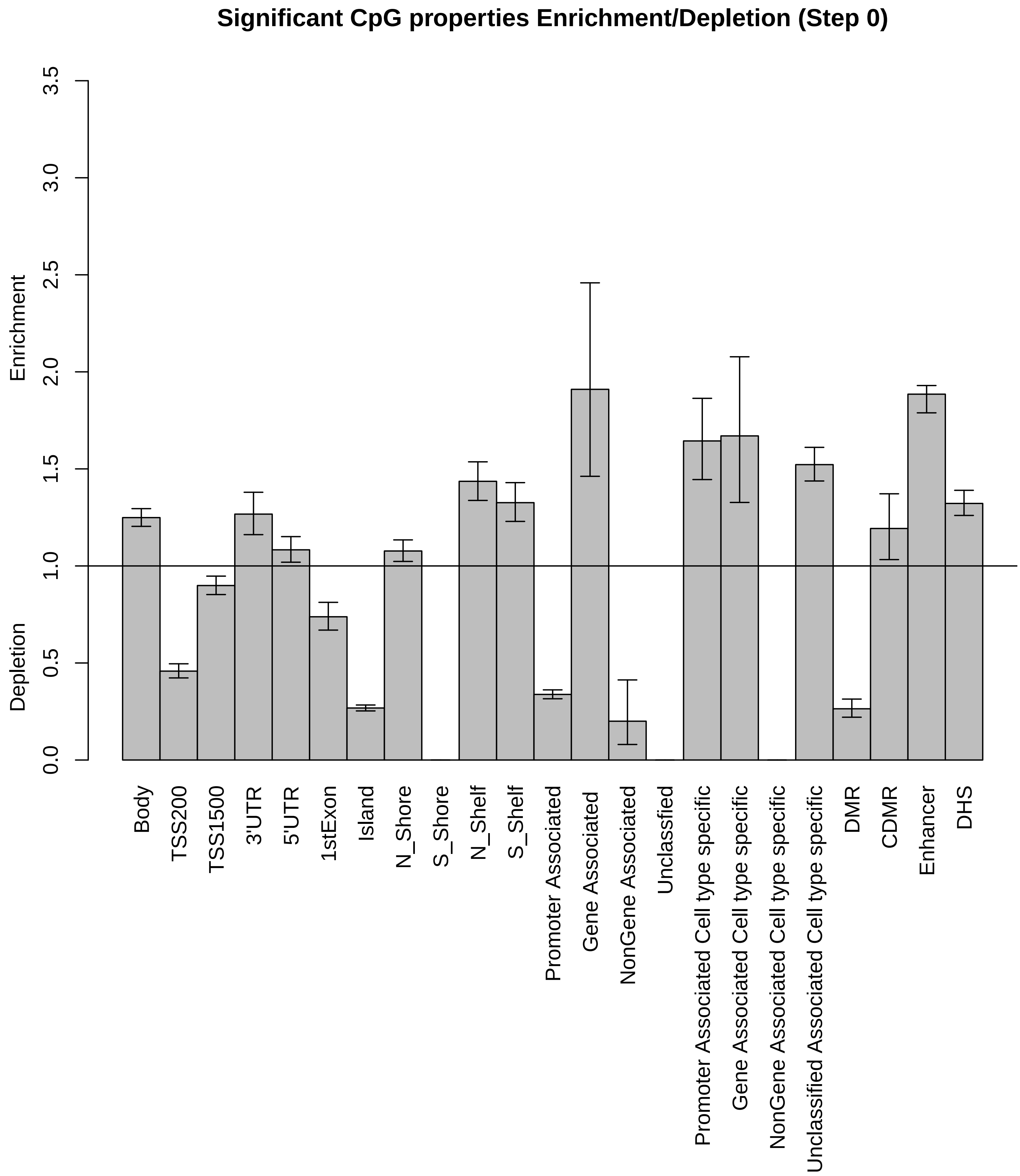

Supplementary Figure 4: Enrichment/depletion barplots for various CpG designations. Enrichment/depletion is computed using the fisher’s exact test and the error bars represent the 95% confidence interval for the odds ratio.

Supplementary Figure 5: Association between NLRC5 methylation (cg07839457) and the urinary 1,3,7,-trimehylurate and urinary neopterin (QMDiab).

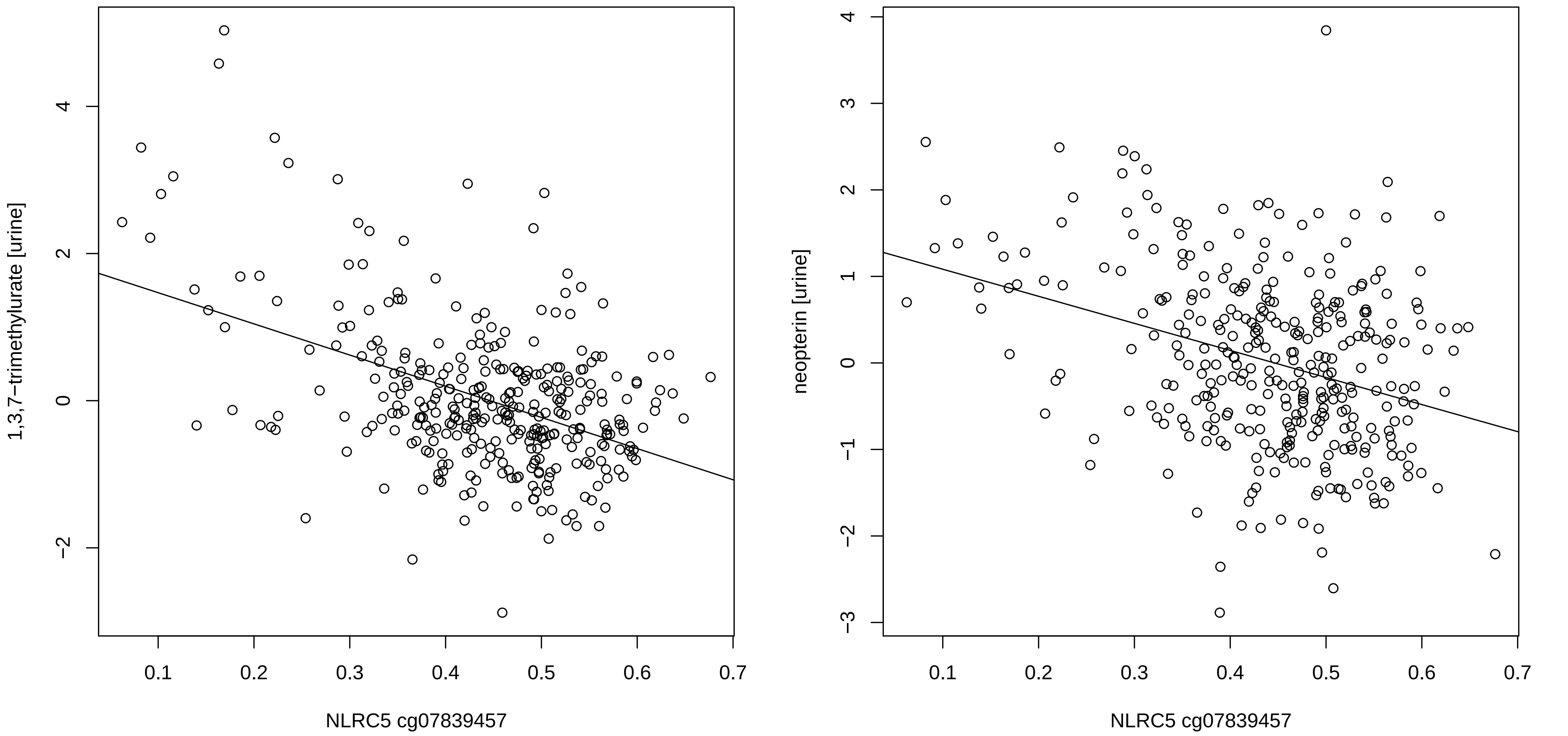

72 CpGs associated with Pappalysin−1 (based on 944 samples)

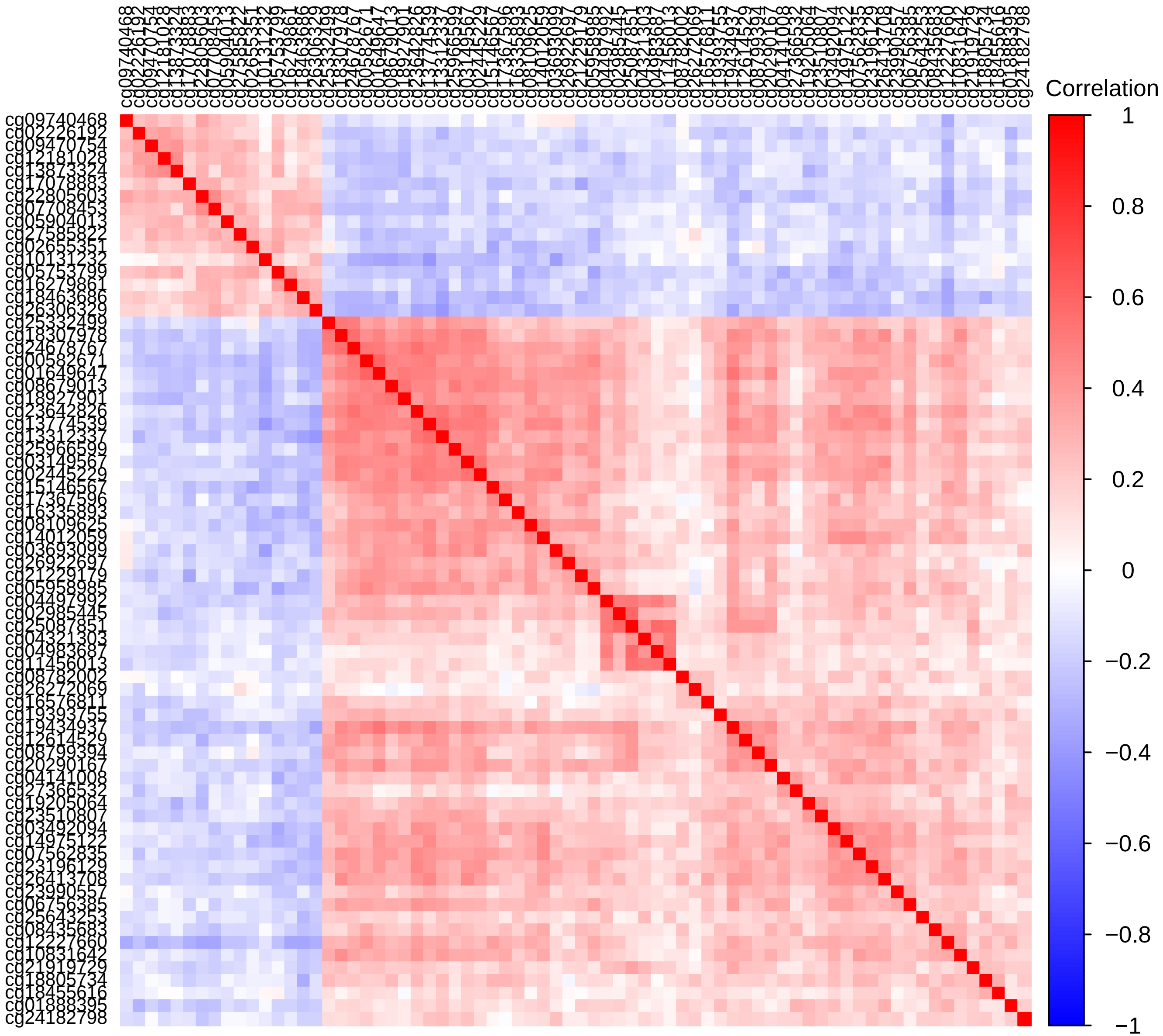

Supplementary Figure 6: Correlation plot of 72 CpG sites associated with pappalysin-1 (PAPPA).

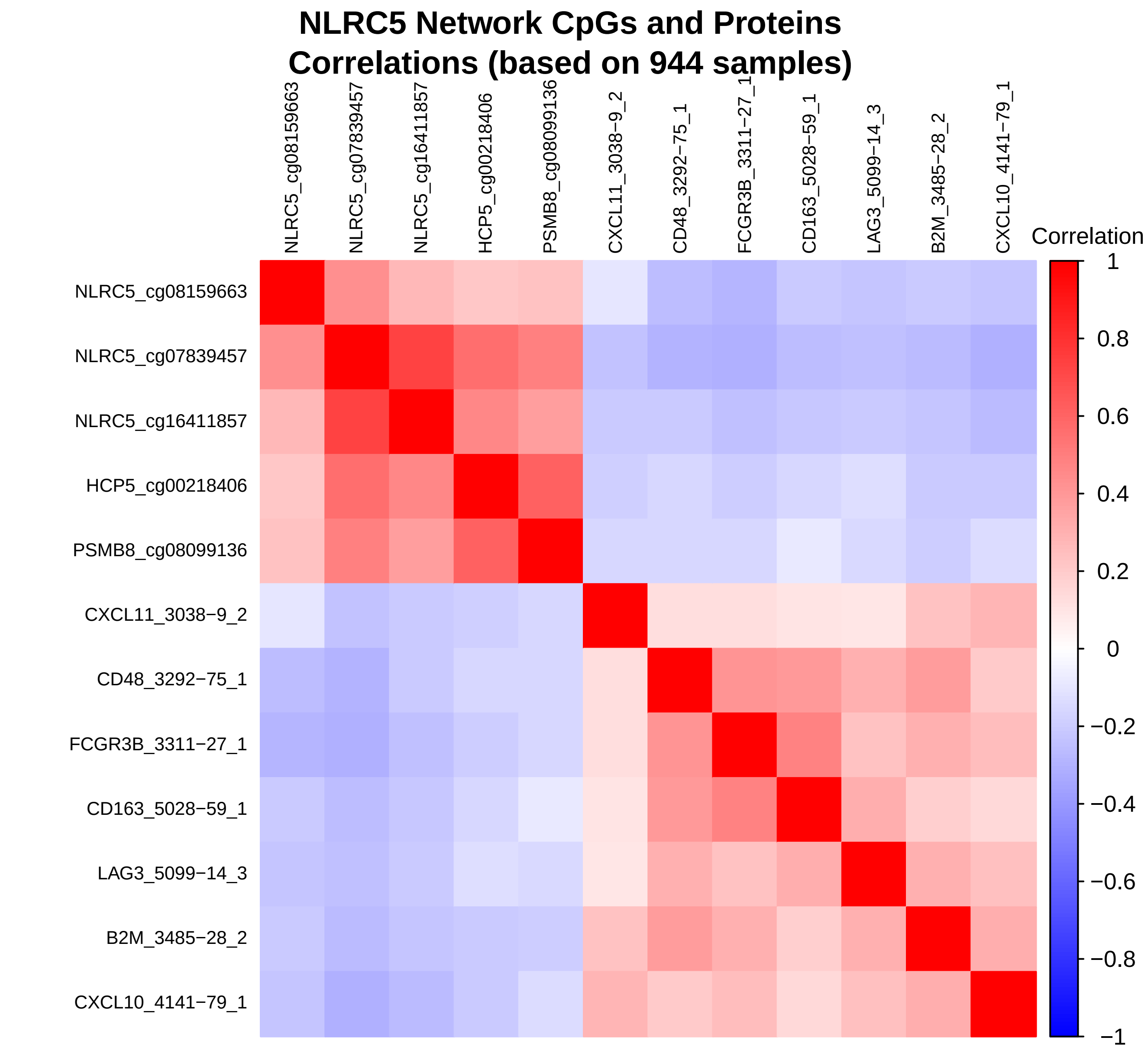

Supplementary Figure 7: Correlation plot of CpG sites and proteins (pQTMs) in the NLRC5 network.

Supplementary Note 1: Replication of previous pEWAS

In a previous EWAS with three Olink panels (cardiovascular, inflammation, and oncology) [1], *NLRC5* methylation (cg07839457) was associated with *CXCL9*, *CXCL11*, *IL-12* and *IL-18*. 121 protein biomarkers were measured in that study, of which we cover 85 (70%) in our panel. Ahsan et al. reported 188 pQTMs (44 proteins and 169 CpG sites) of which we shared 27 proteins (SOMAscan) and are able to attempt replication of 114 pQTMs. We attempted to replicate these 114 pQTMs and only replicated 7 of them (6%) at Bonferroni significance ($p < 0.05/114= 4.39x10^{-4}$), 18 (15.8%) at nominal significance ($p<0.05$) (**Supplementary Data 10**). However, 64 pQTMs (56%) displayed the same trend of association between the two studies. Most importantly, we did replicate the association between C-X-C motif chemokine 11 (*CXCL11*) and 2 *NLRC5* methylation loci (cg07839457 and cg16411857). Our other main associations could not be checked as they were not included in their panel. Although Pappalysin-1 was measured in Ahsan et al., it did not pass QC and was excluded from their study.

Supplementary Note 2: Details of pQTMs eliminated at each step of the pEWAS

Identification of pQTMs driven by gender

After regressing out gender, 2,708 pQTMs (71 proteins and 1,192 CpG sites) were no longer significant. However, in some cases pQTMs became significant due to the correction for a potential confounder. This was in particular the case for gender, where 2,347 new pQTMs reached Bonferroni significance and were replicated. As expected, many of the pQTMs driven by gender implicated sex-related proteins, such as follicle stimulating hormone (*CGA FSHB*; 332 eliminated pQTMs), leptin (*LEP*; 313 pQTMs), human chorionic gonadotropin (*CGA CGB*; 207 pQTMs), and prostate-specific antigen (*KLK3*; 191 pQTMs). The CpG sites of gender-driven pQTMs included previously identified autosomal gender-related sites, such as cg11955727 (chr2:84,105,546; 36 pQTMs), cg20926353 (*TLE1*; 35 pQTMs), and cg00399683 (chr7:153,109,375; 34 pQTLs) [2] [3].

Identification of pQTMs driven by white blood cell composition

After regressing out white blood composition, 12,077 pQTMs (21 proteins and 10,751 CpG sites) were no longer significant. Those pQTMs were dominated by calgranulin B (*S100A9*), a calcium binding protein that is expressed in neutrophils, with 8,495 pQTMs, granzyme A (*GZMA),* a cytotoxic serine protease present in T-lymphocytes, with 2,499 pQTMs, myeloblastin (*PRTN3*) found in polymorphonuclear leukocyte granules, with 528 pQTMs, granulysin (*GNLY*; 315 pQTMs), and matrix metalloproteinase-9 (*MMP9*; 193 pQTLs), among others.

Identification of pQTMs driven by genetic variation

After regressing out genetic variation, 154 pQTMs (62 proteins and 134 CpG sites) were no longer significant. For 90% of these 62 proteins, a genome-wide significant pQTL was already identified in our previous GWAS [4]. In addition, for 43% of these 134 CpG sites, a meQTL was already identified in BIOS [5].

Identification of pQTMs driven by age

Regressing out age eliminated 88 pQTMs (61 CpG sites and 18 proteins). Of these 61 CpG sites, 39 (64%) were previously linked to age in at least one other EWAS [3, 6-9]. Proteins involved in the age-driven pQTMs included A disintegrin and metalloproteinase with thrombospondin motifs 5 (*ADAMTS5*; 37 pQTMs), pleiotrophin (*PTN;* 6 pQTMs), macrophage metalloelastase (*MMP12*; 4 pQTMs), follistatin-related protein 3 (*FSTL3*; 2 pQTMs), and chordin-like protein 1 (*CHRDL1*; 1 pQTM). These proteins were previously associated with chronological age [10]. In KORA, *CHRDL1* and *PTN* are also associated with 12 and 7 of the clock CpG sites identified in Horvarth et al. [6], respectively. Interestingly, these two proteins were also previously identified as the strongest associations with chronological aging in the TwinsUK study using an earlier version of the SOMAscan assay [10]. *CHRDL1* was also significantly associated with birthweight and the individual Framingham 10-year cardiovascular risk score. *PTN* is a secreted growth factor with many functions in multiple tissues and known to be a marker for cardiovascular risk and osteoporosis which are both age related diseases. The age-driven pQTMs also included 11 associations with dermatopontin (*DPT*) and seven with beta-2-microglobulin (*B2M*).

Identification of pQTMs driven by smoking, BMI and diabetes

Regressing out smoking identified 17 pQTMs, (17 CpG sites and 4 proteins). The leading CpG sites were close to the *AHRR* and *F2RL3* genes. Both are known smoking associated methylation sites [11, 12]. Polymeric immunoglobulin receptor (*PIGR*), which we previously identified in association with smoking and CpG methylation of *AHRR* [13], was in eight of the pQTMs driven by smoking. Furthermore, pappalysin-1 (*PAPPA*) was in seven of the pQTMs driven by smoking. Regressing out body mass index excluded two additional pQTMs, one between C-reactive protein (*CRP*) and cg18181703 at *SOCS3*, and the second between platelet glycoprotein Ib alpha chain (*GP1BA*) and cg0008629 at *ROD1.* Both *CRP* and *SOC3* were previously associated body mass index [14, 15]. *GP1BA* is associated with body mass index in KORA ($p=6.66x10^{-6}$) and was also previously linked to atherosclerosis and inflammation in mice [16]. Finally, seven pQTMs were eliminated after regressing out diabetes. These seven pQTMs covered three proteins:

pappalysin-1 (*PAPPA*; 5 pQTMs), beta-2-microglobulin (*B2M*, 1 pQTM) and stem cell growth factor-alpha (*CLEC11A*; 1 pQTM). Two of the CpG sites in the pQTMs that were driven by T2D (cg00851028 and cg16463452) were weakly associated with T2D and HOMA-IR ($p=1.38x10^{-4}$ and 0.0435, respectively) in a previous diabetes EWAS [17].

<u>Supplementary Note 3</u>: Details for all connections in the PAPPA network

KORA clinical phenotypes

Relationship	Details
GABBR1|Alcohol Consumption	See Supplementary Data 8
PAPPA| Alcohol Consumption	See Supplementary Data 7
PAPPA|Metabolic Syndrome	See Supplementary Data 7
PAPPA|Total Cholesterol	See Supplementary Data 7
PAPPA|Triglycerides	See Supplementary Data 7
PAPPA|LDL	See Supplementary Data 7

Disease Association (External Studies)

Relationship	Details	Reference
PAPPA|Cardiovascular Disease	PAPPA is a strong predictor for adverse cardiovascular events in patients with type 2 diabetes.	[18]
PAPPA|Heart Failure	High levels of PAPPA have been shown to be associated with increased risk of heart failure.	[19]
PAPPA|Type 2 Diabetes	PAPPA also is a strong predictor for adverse cardiovascular events in patients with type 2 diabetes.	[18]
PAPPA|Wound Healing	PAPPA also has a role in bone formation, inflammation, wound healing, and female fertility.	[20]
PAPPA|Atherosclerosis	PAPPA is considered a marker of response to injury or diseases such as atherosclerosis or lesion progressio	[21]
PAPPA|Cancer	PAPPA acts as an oncogene, promoting tumor cell proliferation, invasion, and metastasi	[22]

Molecular Association (External Studies)

Relationship	Details	Reference
PRG2|PAPPA	In serum from pregnant human, human pro-PRG2 protein decreases proteolysis activity of human PAPPA protein.	[23]
TNF|PAPPA	In primary culture human dermal fibroblasts, actinomycin D decreases expression of human PAPPA protein that is increased (in a time-dependent and dose-dependent manner) by TNF-alpha [TNF] protein.	[24]
TNF|ELAVL1	Human INTERFERON GAMMA [IFNG] protein in cell culture and human IL1B protein in cell culture and human TNF-alpha [TNF] protein in cell culture decrease expression of human HUR [ELAVL1] mRNA in Dld 1 cells.	[25]
TGFB1|PAPPA	TGF-beta1 [TGFB1] is involved in expression of PAPP-A mRNA.	[26]
PAPPA|ELAVL1	Binding of human ELAVL1 protein and human PAPPA mRNA occurs.	[27]
TGFB1|GABBR1	Transgenic TGF-beta1 [TGFB1] protein is involved in expression of mouse Gabbr1 mRNA in kidney from mouse exhibiting glomerular disease.	[28]
IGF1|TGFB1	In fibroblasts from lung of fetal human, TGFB-1 protein increases expression of human IGF1 mRNA.	[29]
IGF1|TNF	Tumor necrosis factor alpha [TNF] is involved in downregulation of Insulin-like growth factor-1 [IGF1].	[30]
GABBR1|TNF	In POMC neurons from 16 week-old male mouse, homozygous mutant mouse Gabbr1 gene (knockout) increases expression of mouse Tnfa [Tnf] mRNA in hypothalamus from 16 week-old male mouse that involves high fat diet.	[31]
PRG2|TNF	MBP [PRG2] protein increases release of mouse Tnf alpha [Tnf] protein from cultured mast cells from mouse bone marrow that involves rat Scgf [Clec11a] protein.	[32]
CDC42EP4|TNF	In human keratinocytes, human TNF protein increases expression of human CDC42EP4 mRNA.	[33]
SH3PXD2A|ELAVL1	Binding of human ELAVL1 protein and human SH3PXD2A mRNA occurs.	[27]
PRDM2|SOD2	Binding of human PRDM2 protein and human SOD2 protein occurs.	[34]
SOD2|TNF	TNF increases activity of SOD2.	[35]
METRNL|IL10	Circulating METRNL protein increases expression of mouse IL10 mRNA in mouse subcutaneous white adipose tissue.	[36]

IL10|TNF	Uncleavable transmembrane form mutant TNF protein increases production of mouse IL10 protein in mouse tumor derived from 4T1 cells.	[37]

Supplementary Note 4: Details for all connections in the NLRC5 network

KORA clinical phenotypes

Relationship	Details
NLRC5|LDL	See Supplementary Data 8
NLRC5|HDL	See Supplementary Data 8
NLRC5|Total Cholesterol	See Supplementary Data 8
CD48|LDL	See Supplementary Data 7
CD48|Total Cholesterol	See Supplementary Data 7
CD163|HDL	See Supplementary Data 7
CD163|Hypertension	See Supplementary Data 7
CD163|Triglycerides	See Supplementary Data 7
CD163|Metabolic Syndrome	See Supplementary Data 7
CD163|Body Mass Index	See Supplementary Data 7
CD163|Type 2 Diabetes	See Supplementary Data 7
CXCL10|HDL	See Supplementary Data 7
CXCL10|Hypertension	See Supplementary Data 7
CXCL10|Triglycerides	See Supplementary Data 7
FCGR3B|LDL	See Supplementary Data 7
FCGR3B|Total Cholesterol	See Supplementary Data 7
FCGR3B|Hypertension	See Supplementary Data 7
B2M|LDL	See Supplementary Data 7
B2M|HDL	See Supplementary Data 7
B2M|Total Cholesterol	See Supplementary Data 7
B2M|Hypertension	See Supplementary Data 7
B2M|Body Mass Index	See Supplementary Data 7

QMDiab Metabolic Associations

Relationship	Details
NLRC5|neopterin	See Supplementary Data 9
HCP|neopterin	See Supplementary Data 9
PSMB8|neopterin	See Supplementary Data 9
NLRC5|1,3,7-trimethylurate	See Supplementary Data 9
HCP|1,3,7-trimethylurate	See Supplementary Data 9
PSMB8|1,3,7-trimethylurate	See Supplementary Data 9

Disease Association (External Studies)

Relationship	Details	Reference
NLRC5|Cancer	*NLRC5* has been suggested as a promising entry in tumor immunology.	[38] [39]
NLRC5|HIV	*NLRC5* methylation associates with HIV infection.	[40]
NLRC5|Cardiovascular Disease	*NLRC5* methylation associates with circulating IL-18 levels which have been associated to cardiovascular disease. *NLRC5* methylation also associates with Soluble Tumor Necrosis Factor Receptor 2 (sTNFR2) a marker of cardiovascular disease risk in people with diabetes. *NLRC5* methylation is linked to gene expression and inversely associated with the risk of incident coronary heart disease.	[41] [42] [43]
NLRC5|BMI and Obesity	*NLRC5* methylation was shown to be associated with BMI and obesity in Africans.	[44]
NLRC5|Lupus	*NLRC5* methylation associates with lupus.	[42]
NLRC5|Rheumatoid Arthritis	*NLRC5* methylation associates rheumatoid arthritis.	[45]

Other Molecular Association (External Studies)

Relationship	Details	Reference
NLRC5|IL6	Interference of mouse Nlrc5 mRNA by siRNA increases expression of mouse Il6 mRNA in RAW 264.7 cells that is increased by lipopolysaccharide.	[46]
NLRC5|IRF3	NLRC5 ablation reduces MHC class I expression, and enhances IKK and IRF3 phosphorylation in response to TLR stimulation or viral infection	[47]
NLRC5|B2M	Human NLRC5 protein increases expression of human B2M protein in OSE cells that is increased by human RFX5 protein.	[48]
NLRC5|IL10	Mouse Nlrc5 protein is necessary for expression of mouse Il10 mRNA in RAW 264.7 cells that is increased by lipopolysaccharide.	[46]

NLRC5|HLA	Human NLRC5 protein increases expression of human HLA-A mRNA in EBA cells that is increased by human RFXANK isoform b protein.	[48]
NLRC5|MHC Class I	The expression of MHC class I genes is regulated by NLRC5 in coordination with the RFX components through an enhanceosome-dependent manner.<br>It has recently been discovered that another member of the NLR protein family, NLRC5, transcriptionally activates MHC class I genes, and thus acts as CITA (MHC class I transactivator), a counterpart to CIITA.	[49, 50]
PSMB8|IL6	In dendritic cells from human peripheral blood, IL-1beta [IL1B] protein and IL6 protein and TNF-alpha [TNF] protein increase expression of human LMP7 [PSMB8] protein.	[51]
PSMB8|HLA	Repression of human ERAP1 gene by human PRDM1 protein and repression of human MECL1 [PSMB10] gene by human PRDM1 protein and repression of human LMP7 [PSMB8] gene by human PRDM1 protein and repression of human TAPASIN [TAPBP] gene by human PRDM1 protein decrease expression of human Hla-abc protein(s) in cell surface from Hela cells that is increased by IFN gamma [IFNG] protein.	[52]
PSMB8|MCH Class I	Mutant mouse Psmb8 gene (allele $Psmb8^{tm1Hjf}/Psmb8^{tm1Hjf}$) (knockout [homozygous]) in 129P2/OlaHsd mouse decreases level of mouse Mhc class I complex in cell surface.	[53]
PSMB8|NFKB1	Human LMP7 [PSMB8] protein and human LMP2 [PSMB9] protein are involved in production of human p50 [product of NFKB1] protein that is mediated by Proteasome [26s Proteasome] complex.	[54]
NFKB1|IL6	In U937 cells, human IL6 protein increases activity of human NF-kappa-B [NFKB1] protein.	[55]
NFKB1|IRF3	Binding of immobilized human IRF3 protein and p50 [product of NFKB1] protein occurs in a cell-free system.	[56]
NFKB1|IL10	In human mononuclear cells expressing human CD14 protein, benzyl adenine and resiquimod	[57]

	increase induction of human IL10 protein that involves signaling of NF-kB [NFKB1] protein.	
NFKB1|MHC Class I	Interference of human IKKA [CHUK] mRNA by siRNA decreases binding of a DNA fragment containing a NFkB binding site from Mhc Class I gene(s) and a protein complex consisting of human p50 [product of NFKB1] and of human p65 [RELA] in a nuclear extract from Sk Br 3 cells.	[58]
NFKB1|B2M	Binding of promoter fragment containing a NF-kappa B response element from human B2M gene and a protein-protein complex consisting of human p50 [product of NFKB1] and of human C-Rel [REL] and of human p65 [RELA] and of human RELB occurs in a nuclear extracts from MSH cells.	[59]
NFKB1|CXCL11	Interference of human NFKB subunit P105/P50 [NFKB1] mRNA by antisense oligonucleotide decreases production of human CXCL11 protein in cultured keratinocytes treated with human IFNG protein from foreskin of neonatal human that is increased by human IL18 protein.	[60]
NFKB1|CXCL10	P50 [product of NFKB1] protein increases expression of human IP-10 [CXCL10] protein in HepG2 cells.	[61]
B2M|MHC Class I	Binding of human B2M protein and human HLA class I [MHC CLASS I] protein(s) occurs in human platelets.	[62]
B2M|HLA	Binding of human B2M protein and human HLA-A protein and human HLA-F protein occurs.	[63]
B2M|IRF3	IRF3 protein increases transactivation of human B2M gene.	[59]
B2M|IL6	Beta 2-microglobulin [B2M] increases induction of interleukin-6 [IL6]	[64]
IL10|FCGR3A/FCGRCB	In cell surface from isolated peripheral blood monocytes of human, IL10 protein increases expression of human CD16 [FCGR3A] protein.	[65]
IRF3|LAG3	Mouse Irf3 protein is necessary for expression of mouse Lag3 mRNA in conventional dendritic cells expressing mouse Cd11b [Itgam] protein from mouse lung that is increased by house dust mite allergens.	[66]
IL6|LAG3	IL-6 is involved in Expression of LAG-3 mRNA.	[67]
IL10|LAG3	L-10 is involved in Expression of LAG-3 mRNA.	[67]

HLA|LAG3	Mutant mouse Qa-1 [H2-Q1] protein (substitution p.D227K) decreases expression of mouse Lag3 protein in cell surface from spleen effector CD8+ T cells from chronically infected Mus (mouse) infected by LCMV Cl 13.	[68]
IL10|CXCL11	IL10 protein decreases production of I-TAC [CXCL11] protein in neutrophils 2 hours after initial treatment that is increased by LPS [lipopolysaccharide] and Tnf protein(s).	[69]
IRF3|CXCL10	Activation of IRF3 protein by double-stranded DNA increases production of CXCL10 protein.	[70]
IL6|CXCL10	IL6 protein increases production of human IP-10 [CXCL10] protein in cultured Homo sapiens (human) monocyte-derived macrophages that is dependent on phosphorylation of human STAT3 protein.	[71]
IL10|CXCL10	IL10 protein decreases production of IP-10 [CXCL10] protein	[72]
CD163|IL6	The cytokine IL-6 which exerts pro- and anti-inflammatory effects depending on the signaling pathway activated strongly upregulates CD163.	[73]
CD163|IL10	CD163 is induced by IL-10 and glucocorticoids while proinflammatory cytokines like TNF reduce its expression.	[73]
IL6|CD48	In ANBL-6 cells, constitutively active mutant n-Ras protein (unspecified protein mutation) is involved in expression of MEM-102 [CD48] mRNA that involves Il-6 protein.	[74]

Supplementary References

1. Ahsan, M., et al., *The relative contribution of DNA methylation and genetic variants on protein biomarkers for human diseases.* PLoS Genet, 2017. **13**(9): p. e1007005.
2. Martin, E., et al., *Sexual epigenetic dimorphism in the human placenta: implications for susceptibility during the prenatal period.* Epigenomics, 2017. **9**(3): p. 267-278.
3. Zaghlool, S.B., et al., *Association of DNA methylation with age, gender, and smoking in an Arab population.* Clin Epigenetics, 2015. **7**: p. 6.
4. Suhre, K., et al., *Connecting genetic risk to disease end points through the human blood plasma proteome.* Nat Commun, 2017. **8**: p. 14357.
5. Bonder, M.J., et al., *Disease variants alter transcription factor levels and methylation of their binding sites.* Nat Genet, 2017. **49**(1): p. 131-138.
6. Horvath, S., *DNA methylation age of human tissues and cell types.* Genome Biol, 2013. **14**(10): p. R115.
7. Florath, I., et al., *Cross-sectional and longitudinal changes in DNA methylation with age: an epigenome-wide analysis revealing over 60 novel age-associated CpG sites.* Human Molecular Genetics, 2014. **23**(5): p. 1186-1201.
8. Bell, J.T., et al., *Epigenome-Wide Scans Identify Differentially Methylated Regions for Age and Age-Related Phenotypes in a Healthy Ageing Population.* Plos Genetics, 2012. **8**(4): p. 189-200.
9. Bocklandt, S., et al., *Epigenetic Predictor of Age.* Plos One, 2011. **6**(6).
10. Menni, C., et al., *Circulating Proteomic Signatures of Chronological Age.* J Gerontol A Biol Sci Med Sci, 2015. **70**(7): p. 809-16.
11. Breitling, L.P., et al., *Tobacco-Smoking-Related Differential DNA Methylation: 27K Discovery and Replication.* American Journal of Human Genetics, 2011. **88**(4): p. 450-457.
12. Breitling, L.P., et al., *Smoking, F2RL3 methylation, and prognosis in stable coronary heart disease.* Eur Heart J, 2012. **33**(22): p. 2841-8.
13. Zaghlool, S.B., et al., *Deep molecular phenotypes link complex disorders and physiological insult to CpG methylation.* Hum Mol Genet, 2018. **27**(6): p. 1106-1121.
14. Timpson, N.J., et al., *C-reactive protein levels and body mass index: elucidating direction of causation through reciprocal Mendelian randomization.* Int J Obes (Lond), 2011. **35**(2): p. 300-8.
15. Al Muftah, W.A., et al., *Epigenetic associations of type 2 diabetes and BMI in an Arab population.* Clin Epigenetics, 2016. **8**: p. 13.
16. Koltsova, E.K., et al., *Genetic deletion of platelet glycoprotein Ib alpha but not its extracellular domain protects from atherosclerosis.* Thromb Haemost, 2014. **112**(6): p. 1252-63.
17. Kulkarni, H., et al., *Novel epigenetic determinants of type 2 diabetes in Mexican-American families.* Human Molecular Genetics, 2015. **24**(18): p. 5330-5344.
18. Li, W.P., et al., *Pregnancy-associated plasma protein-A is a stronger predictor for adverse cardiovascular outcomes after acute coronary syndrome in type-2 diabetes mellitus.* Cardiovascular Diabetology, 2017. **16**.
19. Dembic, M., et al., *Pregnancy-associated plasma protein-A (PAPP-A) and the proform of the eosinophil major basic protein (ProMBP) are associated with increased risk of death in heart failure patients.* Scand J Clin Lab Invest, 2017. **77**(5): p. 352-357.
20. Conover, C.A., *The IGF-p53 connection in cancer.* Growth Horm IGF Res, 2018. **39**: p. 25-28.
21. Tang, S.L., et al., *PAPP-A negatively regulates ABCA1, ABCG1 and SR-B1 expression by inhibiting LXR alpha through the IGF-I-mediated signaling pathway.* Atherosclerosis, 2012. **222**(2): p. 344-354.

22. Guo, Y., et al., *Pregnancy-associated plasma protein a in cancer: expression, oncogenic functions and regulation.* Am J Cancer Res, 2018. **8**(6): p. 955-963.
23. Overgaard, M.T., et al., *Expression of recombinant human pregnancy-associated plasma protein-A and identification of the proform of eosinophil major basic protein as its physiological inhibitor.* Journal of Biological Chemistry, 2000. **275**(40): p. 31128-31133.
24. Resch, Z.T., et al., *Pregnancy-associated plasma protein A gene expression as a target of inflammatory cytokines.* Endocrinology, 2004. **145**(3): p. 1124-1129.
25. Rodriguez-Pascual, F., et al., *Complex contribution of the 3 '-untranslated region to the expressional regulation of the human inducible nitric-oxide synthase gene - Involvement of the RNA-binding protein HuR.* Journal of Biological Chemistry, 2000. **275**(34): p. 26040-26049.
26. Gonzalez, C., et al., *Transforming growth factor-beta 1 modulates insulin-like growth factor binding protein-4 expression and proteolysis in cultured periosteal explants.* Growth Hormone & Igf Research, 2010. **20**(2): p. 81-86.
27. Abdelmohsen, K., et al., *Ubiquitin-mediated proteolysis of HuR by heat shock.* Embo Journal, 2009. **28**(9): p. 1271-1282.
28. Ju, W.J., et al., *Renal Gene and Protein Expression Signatures for Prediction of Kidney Disease Progression (vol 174, pg 2073, 2009).* American Journal of Pathology, 2009. **175**(3): p. 1349-1349.
29. Chambers, R.C., et al., *Global expression profiling of fibroblast responses to transforming growth factor-beta1 reveals the induction of inhibitor of differentiation-1 and provides evidence of smooth muscle cell phenotypic switching.* Am J Pathol, 2003. **162**(2): p. 533-46.
30. Braun, A., et al., *Selective regulation of growth factor expression in cultured cortical astrocytes by neuro-pathological toxins.* Neurochemistry International, 2009. **55**(7): p. 610-618.
31. Ito, Y., et al., *GABA Type B Receptor Signaling in Proopiomelanocortin Neurons Protects Against Obesity, Insulin Resistance, and Hypothalamic Inflammation in Male Mice on a High-Fat Diet.* Journal of Neuroscience, 2013. **33**(43): p. 17166-17173.
32. Furuta, G.T., et al., *Stem cell factor influences mast cell mediator release in response to eosinophil-derived granule major basic protein.* Blood, 1998. **92**(3): p. 1055-1061.
33. Banno, T., A. Gazel, and M. Blumenberg, *Effects of tumor necrosis factor-alpha (TNF alpha) in epidermal keratinocytes revealed using global transcriptional profiling.* Journal of Biological Chemistry, 2004. **279**(31): p. 32633-32642.
34. Miyamoto-Sato, E., et al., *A comprehensive resource of interacting protein regions for refining human transcription factor networks.* PLoS One, 2010. **5**(2): p. e9289.
35. Karube-Harada, A., et al., *Induction of manganese superoxide dismutase by tumour necrosis factor-alpha in human endometrial stromal cells.* Molecular Human Reproduction, 2001. **7**(11): p. 1065-1072.
36. Rao, R.R., et al., *Meteorin-like Is a Hormone that Regulates Immune-Adipose Interactions to Increase Beige Fat Thermogenesis.* Cell, 2014. **157**(6): p. 1279-1291.
37. Hu, X., et al., *Transmembrane TNF-alpha Promotes Suppressive Activities of Myeloid-Derived Suppressor Cells via TNFR2.* Journal of Immunology, 2014. **192**(3): p. 1320-1331.
38. Chelbi, S.T. and G. Guarda, *NLRC5, a promising new entry in tumor immunology.* Journal for Immunotherapy of Cancer, 2016. **4**.
39. Yoshihama, S., et al., *NLRC5/CITA: A Key Player in Cancer Immune Surveillance.* Trends in Cancer, 2017. **3**(1): p. 28-38.
40. Zhang, X.Y., et al., *Epigenome-wide differential DNA methylation between HIV-infected and uninfected individuals.* Epigenetics, 2016. **11**(10): p. 750-760.
41. Zeller, T., et al., *Molecular Characterization of the NLRC4 Expression in Relation to Interleukin-18 Levels.* Circ Cardiovasc Genet, 2015. **8**(5): p. 717-26.

42. Mendelson, M.M., et al., *Epigenome-Wide Association Study of Soluble Tumor Necrosis Factor Receptor 2 Levels in the Framingham Heart Study.* Frontiers in Pharmacology, 2018. **9**.
43. Aslibekyan, S., et al., *Association of Methylation Signals With Incident Coronary Heart Disease in an Epigenome-Wide Assessment of Circulating Tumor Necrosis Factor alpha.* Jama Cardiology, 2018. **3**(6): p. 463-472.
44. Meeks, K.A.C., et al., *An epigenome-wide association study in whole blood of measures of adiposity among Ghanaians: the RODAM study.* Clin Epigenetics, 2017. **9**: p. 103.
45. Zou, J., et al., *Epigenome-wide association studies without the need for cell-type composition.* Nature Methods, 2014. **11**(3): p. 309-U283.
46. Benko, S., et al., *NLRC5 limits the activation of inflammatory pathways.* J Immunol, 2010. **185**(3): p. 1681-91.
47. Tong, Y., et al., *Enhanced TLR-induced NF-kappaB signaling and type I interferon responses in NLRC5 deficient mice.* Cell Res, 2012. **22**(5): p. 822-35.
48. Meissner, T.B., et al., *NLRC5 cooperates with the RFX transcription factor complex to induce MHC class I gene expression.* J Immunol, 2012. **188**(10): p. 4951-8.
49. Yao, Y. and Y. Qian, *Expression regulation and function of NLRC5.* Protein Cell, 2013. **4**(3): p. 168-75.
50. Meissner, T.B., A. Li, and K.S. Kobayashi, *NLRC5: a newly discovered MHC class I transactivator (CITA).* Microbes Infect, 2012. **14**(6): p. 477-84.
51. Macagno, A., et al., *Dendritic cells up-regulate immunoproteasomes and the proteasome regulator PA28 during maturation.* Eur J Immunol, 1999. **29**(12): p. 4037-42.
52. Doody, G.M., et al., *PRDM1/BLIMP-1 modulates IFN-gamma-dependent control of the MHC class I antigen-processing and peptide-loading pathway.* J Immunol, 2007. **179**(11): p. 7614-23.
53. Kincaid, E.Z., et al., *Mice completely lacking immunoproteasomes show major changes in antigen presentation.* Nature Immunology, 2012. **13**(2): p. 129-135.
54. Hayashi, T. and D. Faustman, *Essential role of human leukocyte antigen-encoded proteasome subunits in NF-kappaB activation and prevention of tumor necrosis factor-alpha-induced apoptosis.* J Biol Chem, 2000. **275**(7): p. 5238-47.
55. Haugen, F. and C.A. Drevon, *Activation of nuclear factor-kappaB by high molecular weight and globular adiponectin.* Endocrinology, 2007. **148**(11): p. 5478-86.
56. Yang, H.M., et al., *Mechanism for transcriptional synergy between interferon regulatory factor (IRF)-3 and IRF-7 in activation of the interferon-beta gene promoter.* European Journal of Biochemistry, 2004. **271**(18): p. 3693-3703.
57. Chinen, J. and W.T. Shearer, *Advances in basic and clinical immunology in 2006.* J Allergy Clin Immunol, 2007. **120**(2): p. 263-70.
58. Merkhofer, E.C., P. Cogswell, and A.S. Baldwin, *Her2 activates NF-kappaB and induces invasion through the canonical pathway involving IKKalpha.* Oncogene, 2010. **29**(8): p. 1238-48.
59. Gobin, S.J., P. Biesta, and P.J. Van den Elsen, *Regulation of human beta 2-microglobulin transactivation in hematopoietic cells.* Blood, 2003. **101**(8): p. 3058-64.
60. Kanda, N., et al., *IL-18 enhances IFN-gamma-induced production of CXCL9, CXCL10, and CXCL11 in human keratinocytes.* European Journal of Immunology, 2007. **37**(2): p. 338-350.
61. Zhou, Y., et al., *Hepatitis B virus protein X-induced expression of the CXC chemokine IP-10 is mediated through activation of NF-kappaB and increases migration of leukocytes.* J Biol Chem, 2010. **285**(16): p. 12159-68.
62. Morales, P.J., et al., *Placental cell expression of HLA-G2 isoforms is limited to the invasive trophoblast phenotype.* J Immunol, 2003. **171**(11): p. 6215-24.
63. Goodridge, J.P., et al., *HLA-F Complex without Peptide Binds to MHC Class I Protein in the Open Conformer Form.* Journal of Immunology, 2010. **184**(11): p. 6199-6208.

64. Iida, Y., et al., *Beta 2-microglobulin modified with advanced glycation end products induces interleukin-6 from human macrophages: role in the pathogenesis of hemodialysis-associated amyloidosis.* Biochem Biophys Res Commun, 1994. **201**(3): p. 1235-41.
65. Iwahashi, M., et al., *Expression of Toll-like receptor 2 on CD16+ blood monocytes and synovial tissue macrophages in rheumatoid arthritis.* Arthritis Rheum, 2004. **50**(5): p. 1457-67.
66. Janss, T., et al., *Interferon response factor-3 promotes the pro-Th2 activity of mouse lung CD11b(+) conventional dendritic cells in response to house dust mite allergens.* Eur J Immunol, 2016. **46**(11): p. 2614-2628.
67. Matsuzaki, J., et al., *Tumor-infiltrating NY-ESO-1-specific CD8+ T cells are negatively regulated by LAG-3 and PD-1 in human ovarian cancer.* Proc Natl Acad Sci U S A, 2010. **107**(17): p. 7875-80.
68. Holderried, T.A., et al., *Genetic disruption of CD8+ Treg activity enhances the immune response to viral infection.* Proc Natl Acad Sci U S A, 2013. **110**(52): p. 21089-94.
69. Moore, K.W., et al., *Interleukin-10 and the interleukin-10 receptor.* Annu Rev Immunol, 2001. **19**: p. 683-765.
70. Zitvogel, L., O. Kepp, and G. Kroemer, *Decoding Cell Death Signals in Inflammation and Immunity.* Cell, 2010. **140**(6): p. 798-804.
71. Xu, W., et al., *Macrophages induce differentiation of plasma cells through CXCL10/IP-10.* J Exp Med, 2012. **209**(10): p. 1813-23, S1-2.
72. Couper, K.N., D.G. Blount, and E.M. Riley, *IL-10: the master regulator of immunity to infection.* J Immunol, 2008. **180**(9): p. 5771-7.
73. Buechler, C., K. Eisinger, and S. Krautbauer, *Diagnostic and prognostic potential of the macrophage specific receptor CD163 in inflammatory diseases.* Inflamm Allergy Drug Targets, 2013. **12**(6): p. 391-402.
74. Croonquist, P.A., et al., *Gene profiling of a myeloma cell line reveals similarities and unique signatures among IL-6 response, N-ras-activating mutations, and coculture with bone marrow stromal cells.* Blood, 2003. **102**(7): p. 2581-2592.
