# Supplementary material for: Epigenetics meets proteomics in an epigenome-wide association study with circulating blood plasma protein traits
Source: Nat Commun. 2020 Jan 3;11:15. doi: 10.1038/s41467-019-13831-w (PMC6941977; doi:10.1038/s41467-019-13831-w)
Supplement: Supplementary file 14 — Description of Additional Supplementary Files [file 41467_2019_13831_MOESM14_ESM.pdf]

Title: Supplementary Data 1:
Description: Annotation of the SOMAmer probes.

Title: Supplementary Data 2:
Description: Replicated pQTMs identified at the different steps of the pEWAS.

Title: Supplementary Data 3:
Description: pQTMs identified and replicated at the different EWAS steps.

Title: Supplementary Data 4:
Description: pEWAS results – 98 replicated CpG-protein associations (pQTMs) after various regression steps.

Title: Supplementary Data 5:
Description: Enrichment of pQTMs for various CpG site characteristics.

Title: Supplementary Data 6:
Description: EWAS atlas lookup of the 98 final pQTMs.

Title: Supplementary Data 7:
Description: Clinical phenotypes associated with pEWAS proteins (KORA data).

Title: Supplementary Data 8:
Description: Clinical phenotypes associated with pEWAS CpGs (KORA data).

Title: Supplementary Data 9:
Description: Metabolites associated with pQTM CpG sites (QMDiab data).

Title: Supplementary Data 10:
Description: Replication of previously reported pQTMs (Ahsan *et al*. [7]).

Title: Supplementary Data 11:
Description: NLRC5 (cg07839457) to protein associations (KORA) at relaxed significance level (p=0.05/1,123).

Title: Supplementary Data 12:
Description: Comparison of our successive residuals method to a joint one-step regression.

مؤسسة قطر - المدينة التعليمية، ص.ب: ٢٤١٤٤ - الدوحة، قطر   هاتف | ٨٢٠٠ ٤٤٩٢ ٩٧٤+   فاكس | ٨٢٢٢ ٤٤٩٢ ٩٧٤+   www.qatar-weill.cornell.edu
Qatar Foundation - Education City, P.O. Box: 24144 - Doha, Qatar   Tel | +974 4492 8200   Fax | +974 4492 8222   www.qatar-weill.cornell.edu
