# Supplementary material for: Epigenetics meets proteomics in an epigenome-wide association study with circulating blood plasma protein traits
Source: Nat Commun. 2020 Jan 3;11:15. doi: 10.1038/s41467-019-13831-w (PMC6941977; doi:10.1038/s41467-019-13831-w)
Supplement: Supplementary file 15 — Reporting Summary [file 41467_2019_13831_MOESM15_ESM.pdf]

nature research

Corresponding author(s): Karsten Suhre

Reporting Summary

Nature Research wishes to improve the reproducibility of the work that we publish. This form provides structure for consistency and transparency in reporting. For further information on Nature Research policies, see Authors & Referees and the Editorial Policy Checklist.

Statistical parameters

When statistical analyses are reported, confirm that the following items are present in the relevant location (e.g. figure legend, table legend, main text, or Methods section).

n/a	Confirmed	
☐	☒	The exact sample size (n) for each experimental group/condition, given as a discrete number and unit of measurement
☒	☐	An indication of whether measurements were taken from distinct samples or whether the same sample was measured repeatedly
☐	☒	The statistical test(s) used AND whether they are one- or two-sided<br>*Only common tests should be described solely by name; describe more complex techniques in the Methods section.*
☐	☒	A description of all covariates tested
☐	☒	A description of any assumptions or corrections, such as tests of normality and adjustment for multiple comparisons
☐	☒	A full description of the statistics including central tendency (e.g. means) or other basic estimates (e.g. regression coefficient) AND variation (e.g. standard deviation) or associated estimates of uncertainty (e.g. confidence intervals)
☒	☐	For null hypothesis testing, the test statistic (e.g. F, t, r) with confidence intervals, effect sizes, degrees of freedom and P value noted<br>*Give P values as exact values whenever suitable.*
☒	☐	For Bayesian analysis, information on the choice of priors and Markov chain Monte Carlo settings
☒	☐	For hierarchical and complex designs, identification of the appropriate level for tests and full reporting of outcomes
☐	☒	Estimates of effect sizes (e.g. Cohen's d, Pearson's r), indicating how they were calculated
☒	☐	Clearly defined error bars<br>*State explicitly what error bars represent (e.g. SD, SE, CI)*

Our web collection on statistics for biologists may be useful.

Software and code

Policy information about availability of computer code

Data collection	no specific software was used
Data analysis	R version 3.5

For manuscripts utilizing custom algorithms or software that are central to the research but not yet described in published literature, software must be made available to editors/reviewers upon request. We strongly encourage code deposition in a community repository (e.g. GitHub). See the Nature Research guidelines for submitting code & software for further information.

Data

Policy information about availability of data

All manuscripts must include a data availability statement. This statement should provide the following information, where applicable:

- Accession codes, unique identifiers, or web links for publicly available datasets
- A list of figures that have associated raw data
- A description of any restrictions on data availability

Summary data of all significant associations is provided in the supplementary tables. The informed consent given by the study participants does not cover posting of participant level phenotype and genotype data in public databases. However, data are available upon request from KORA-gen (http://epi.helmholtz-muenchen.de/kora-gen). Requests are submitted online and are subject to approval by the KORA board.

Field-specific reporting

Please select the best fit for your research. If you are not sure, read the appropriate sections before making your selection.

☒ Life sciences ☐ Behavioural & social sciences ☐ Ecological, evolutionary & environmental sciences

For a reference copy of the document with all sections, see nature.com/authors/policies/ReportingSummary-flat.pdf

Life sciences study design

All studies must disclose on these points even when the disclosure is negative.

Sample size	Sample size was determined by the available financial resources. A-posteriori power calculations we conducted to estimate replication power.
Data exclusions	All samples that passed standard platform QC for methylation, genotyping, and proteomics were used.
Replication	An independent replication cohort was used. The fraction of replicated pQTMs is reported (Table 1) and discussed.
Randomization	As this study only involves participants from the general population, no randomization was required.
Blinding	Blinding was not relevant as no groups were defined.

Reporting for specific materials, systems and methods

Materials & experimental systems

n/a	Involved in the study	
☒	☐	Unique biological materials
☒	☐	Antibodies
☒	☐	Eukaryotic cell lines
☒	☐	Palaeontology
☒	☐	Animals and other organisms
☐	☒	Human research participants

Methods

n/a	Involved in the study	
☒	☐	ChIP-seq
☒	☐	Flow cytometry
☒	☐	MRI-based neuroimaging

Human research participants

Policy information about studies involving human research participants

Population characteristics	The KORA F4 study is a population-based cohort of 3,080 subjects living in the southern Germany. Study participants were recruited between 2006 and 2008 comprising individuals with age ranging from 32 to 81. The Qatar Metabolomics Study on Diabetes (QMDiab) is a cross-sectional case-control study that was carried out in 2012 at the Dermatology Department in Hamad Medical Corporation (HMC Doha, Qatar). This study has been described previously and comprises 388 study participants from Arab and Asian ethnicities.
Recruitment	No recruitment was conducted for this study. Here we only analyze anonymized data from previous studies, deemed non-human subject research
